# Arthrinins E–G, Three Botryane Sesquiterpenoids from the Plant Endophytic Fungus *Arthrinium* sp. HS66

**DOI:** 10.1007/s13659-020-00248-y

**Published:** 2020-07-12

**Authors:** Xiao-Zheng Su, Jian-Wei Tang, Kun Hu, Xiao-Nian Li, Han-Dong Sun, Pema-Tenzin Puno

**Affiliations:** 1 State Key Laboratory of Phytochemistry and Plant Resources in West China, Kunming Institute of Botany, Chinese Academy of Sciences, Kunming, 650201 People’s Republic of China; 2 Yunnan Key Laboratory of Natural Medicinal Chemistry, Kunming Institute of Botany, Chinese Academy of Sciences, Kunming, 650201 People’s Republic of China; 3 University of Chinese Academy of Sciences, Beijing, 100049 People’s Republic of China

**Keywords:** *Isodon xerophilus*, Endophytic fungus, *Arthrinium*, Botryane sesquiterpenoid, Quantum chemical calculation

## Abstract

Arthrinins E–G (**1**–**3**), three new sesquiterpenoids possessing non-isoprenoid botryane skeleton, were isolated from the fermentation of an endophytic fungus named *Arthrinium* sp. HS66 which colonized in the stems of *Isodon xerophilus*. Their structures were determined by extensive spectroscopic methods. Furthermore, the structure of **1** was unambiguously confirmed by X-ray diffraction, while those of **2** and **3** were verified through quantum chemical calculation of NMR data and ECD spectra.

**Electronic supplementary material:**

The online version of this article (doi:10.1007/s13659-020-00248-y) contains supplementary material, which is available to authorized users.

## Introduction

Fungal metabolites have received considerable attention nowadays because their diverse chemical structures and bioactivities greatly facilitate the progress of drug discovery [1]. Botryanes are a class of fungus-derived sesquiterpene metabolites featuring characteristic bicyclic non-isoprenoid system [2–4], and they were found to possess broad spectrum of biological activities, such as cytotoxicity[5, 6], phytotoxicity [7, 8], and antimicrobial property [9, 10]. Furthermore, fascinating by the botryane sesquiterpenoids, researchers have conducted plenty of in-depth investigations about their structure–activity relationships [11], synthesis [12, 13] and biosynthetic pathway [14–16].

**Fig. 1:**
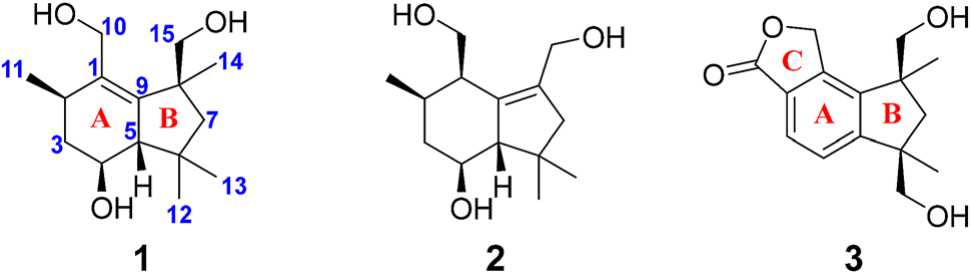
Chemical structures of compounds **1**–**3**

Fungal endophytes have become an important source for discovering structurally novel and biologically active secondary metabolites [17]. Over the past several years, our groups have made great efforts to study the secondary metabolites from endophytic fungi inhabiting the *Isodon* species. As a result, isopenicin A, a potent inhibitor of Wnt signaling [18], as well as several antineoplastic compounds like phomopchalasins A and B [19] have been successfully obtained. In the present research, an endophytic fungus colonizing in the stems of *Isodon xerophilus* was discovered and identified as *Arthrinium* sp. HS66. Subsequent large fermentation and chemical investigation on this strain resulted in the isolation of three new botryane sesquiterpenoids named arthrinins E–G (**1**–**3**). Notably, compound **2** possesses uncommon 15-nor-botryane skeleton. Herein, details of the isolation, structure elucidation, and cytotoxicity of these compounds were reported (Fig. 1).

## Results and Discussion

Compound **1** was isolated as colorless oil, and it was assigned a molecular formula of C_15_H_26_O_3_ according to its positive HRESIMS ion peak at *m/z* 277.1775 ([M + Na]^+^, calcd for 277.1774), which required three degrees of unsaturation. The IR spectrum showed the presence of hydroxyl (3374 cm^–1^) group. The ^1^H NMR data (Table 1) exhibited the existence of three singlet methyls (*δ*_H_ 0.97, 1.18 and 1.38), one doublet methyl (*δ*_H_ 1.10, d, *J* = 6.9 Hz), a pair of nonequivalent oxygenated methylene protons at *δ*_H_ 4.04 (dd, *J* = 11.9, 2.1 Hz)/4.33 (d, *J* = 11.9 Hz), one hydroxylated methylene signal at *δ*_H_ 3.30 (overlap), and one hydroxylated methine proton at *δ*_H_ 3.63 (ddd, *J* = 11.9, 9.6, 3.6 Hz). The analysis of its ^13^C NMR and DEPT spectra revealed 15 carbon resonances which were assigned as four methyls, four *sp*^3^ methylenes, three *sp*^3^ methines, two *sp*^3^ quaternary carbons and two olefinic carbons (Table 2). These data suggested that compound **1** might be a botryane sesquiterpenoid [20].

**Table 1:** ^1^H NMR data (*δ* in ppm, *J* in Hz) of compounds **1**–**3**

No.	**1**^**a**^	**2**^**a**^	**3**^**b**^
	*δ*_H_, mult (*J*)	*δ*_H_, mult (*J*)	*δ*_H_, mult (*J*)
1		2.60 (dt, 10.2, 4.4)	
2	2.58 (m)	1.67 (overlap)	
3	*β* 1.97 (ddd, 12.0, 5.8, 3.6)	*β* 1.31 (m)	7.72 (d, 7.9)
	*α* 1.29 (overlap)	*α* 1.67 (overlap)	
4	3.63 (ddd, 11.9, 9.6, 3.6)	3.57 (td, 10.8, 4.5)	7.43 (d, 7.9)
5	2.10 (ddd, 9.6, 3.5, 2.1)	2.30 (d, 10.8)	
6			
7	*β* 1.74 (d, 13.0)	*β* 2.37 (dd, 15.6,2.3)	*β* 2.36 (d, 13.9)
	*α* 1.30 (overlap)	*α* 2.16 (dd, 15.6, 2.3)	*α* 1.70 (d, 13.9)
8			
9			
10	4.33 (d, 11.9)	3.69 (dd, 10.2, 4.4)	5.55 (d, 15.7)
	4.04 (dd, 11.9, 2.1)	3.45 (t, 10.2)	5.46 (d, 15.7)
11	1.10 (d, 6.9)	1.03 (d, 7.0)	
12	0.97 (s)	1.07 (s)	3.61 (d, 10.8)
			3.58 (d, 10.8)
13	1.18 (s)	1.23 (s)	1.34 (s)
14	1.38 (s)	4.05 (s)	1.37 (s)
15	3.30 (overlap)		3.64 (s)

^a^Recorded at 600 MHz, Recorded in CD_3_OD

^b^Recorded at 500 MHz, Recorded in CD_3_OD

**Table 2:** ^13^C NMR data (*δ* in ppm) of compounds **1**–**3**

No.	**1**^**a**^	**2**^**a**^	**3**^**b**^
	*δ*_C_, type	*δ*_C_, type	*δ*_C_, type
1	136.0, C	43.5, CH	145.2, C
2	34.4, CH	33.7, CH	126.0, C
3	44.1, CH_2_	40.8, CH_2_	125.4, CH
4	70.0, CH	71.9, CH	126.1, CH
5	61.4, CH	60.9, CH	157.8, C
6	40.0, C	39.7, C	50.1, C
7	56.7, CH_2_	51.7, CH_2_	47.8, CH_2_
8	47.5, C	136.9, C	50.2, C
9	145.9, C	139.8, C	144.7, C
10	58.9, CH_2_	59.9, CH_2_	71.1, CH_2_
11	20.7, CH_3_	19.4, CH_3_	173.9, C
12	23.9, CH_3_	25.6, CH_3_	71.3, CH_2_
13	30.9, CH_3_	31.6, CH_3_	27.2, CH_3_
14	26.1, CH_3_	59.2, CH_2_	24.9, CH_3_
15	72.6, CH_2_		70.8, CH_2_

^a^Recorded at 150 MHz, Recorded in CD_3_OD

^b^Recorded at 125 MHz, Recorded in CD_3_OD

**Fig. 2:**
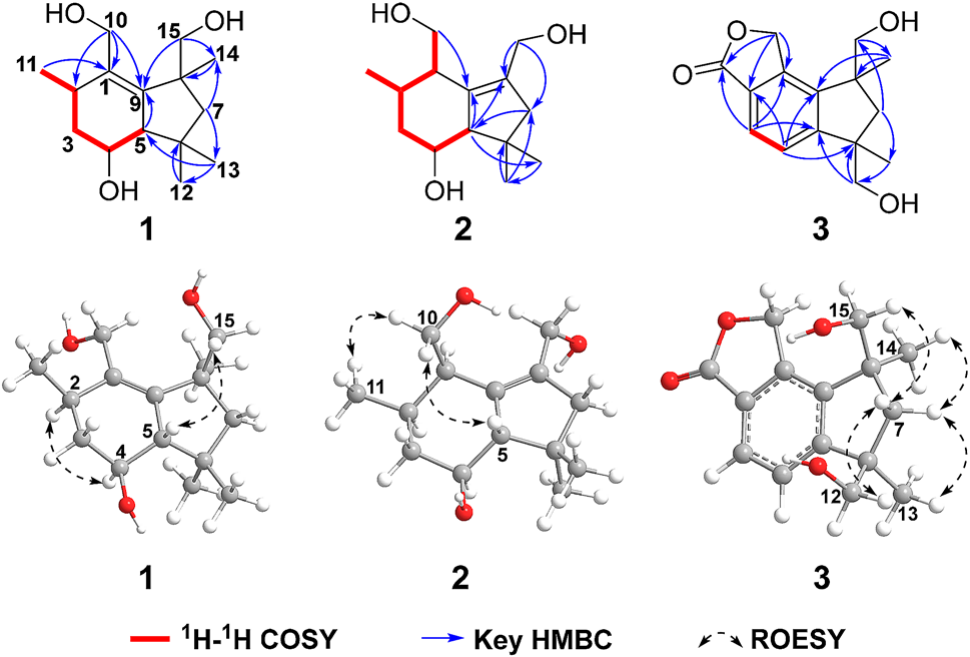
Key 2D NMR correlations of compounds **1**–**3**

Specifically, a structural subunit of C-11/C-2/C-3/C-4/C-5 could be established as an isolated spin-system according to the ^1^H-^1^H COSY correlations of H_3_-11/H-2/H_2_-3/H-4/H-5. Then, the subunit can be assigned to ring A through the HMBC correlations from H-5 to C-9 (*δ*_C_ 145.9), from H_2_-10 to C-1 (*δ*_C_ 136.0), C-2 (*δ*_C_ 34.4) and C-9, from H_3_-11 to C-1. Meanwhile, the HMBC correlations from H_2_-7 to C-13 (*δ*_C_ 30.9) and C-14 (*δ*_C_ 26.1), from H_3_-12 to C-6 (*δ*_C_ 40.0), from H_3_-13 to C-5 (*δ*_C_ 61.4) and C-12 (*δ*_C_ 23.9), from H_3_-14 to C-8 (*δ*_C_ 47.5), from H_2_-15 to C-9 and C-14, in combination with the remaining one degree of hydrogen deficiency, successfully established the ring B as well as its connection with ring A (Fig. 2). Hence, the planar structure of compound **1** was resolved.

**Fig. 3:**
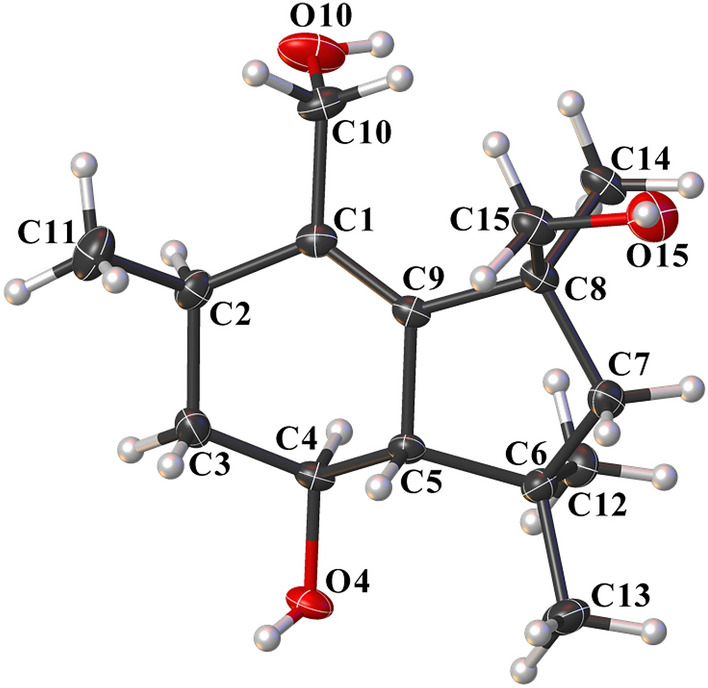
X-ray crystallographic structure of **1**

The relative configuration of **1** was deduced by analyses of ROESY and ^1^H NMR data. Randomly assigning H-2 as *α*-oriented, the cross peaks of H-4/H-2 and H-5/H_2_-15, together with the coupling constant (*J* = 9.6 Hz) between H-4 and H-5 indicated that both H-4 and CH_3_-14 adopted *α*-orientation, while both H-5 and CH_2_OH-15 adopted *β*-orientation (Fig. 2). Fortunately, colorless square crystals of **1** were eventually obtained through slow evaporation of methanol. Then a single-crystal X-ray diffraction experiment with Cu K*α* radiation of **1** [Flack parameter = 0.07(3)] unambiguously verified the aforementioned deduction and assigned the absolute configuration of **1** as 2*R*,4*S*,5*S*, and 8*S* (Fig. 3). Arthrinins A–D from the sponge derived fungus *Arthrinium* sp. has been reported, so we gave compound **1** the trivial name from arthrinin E [21].

Compound **2** was obtained as colorless oil. Its molecular formula C_14_H_24_O_3_ was ascertained by the positive HRESIMS ion peak (*m/z* 263.1618, [M + Na]^+^, calcd for 263.1618), indicating three degrees of unsaturation. Comparison of the ^1^H NMR spectrum of **2** with that of **1** disclosed that **2** was structurally analogous to compound **1** (Table 1). Furthermore, the ^13^C NMR and DEPT spectra, which demonstrated 14 carbons resonances involving three methyls, four *sp*^3^ methylenes, four *sp*^3^ methines, one quaternary carbon and two olefinic carbons (Table 2), suggested that the structure of **2** was highly similar to that of boledulin C [22], a 15-nor-botryane sesquiterpenoid.

More credible evidence was obtained from 2D NMR spectra (Figs. S20-23) of **2**. On one hand, the structure of ring A was established by the ^1^H-^1^H COSY correlations of H_2_-10/H-1/H-2/H_2_-3/H-4/H-5 and H-2/H_3_-11 and HMBC correlations from H-5 to C-9 (*δ*_C_ 139.8), from H_2_-10 to C-9. On the other hand, the HMBC correlations from H-5 to C-8 (*δ*_C_ 136.9) and C-13 (*δ*_C_ 31.6), from H_2_-7 to C-5 (*δ*_C_ 60.9), from H_3_-12 to C-6 (*δ*_C_ 39.7) and C-7 (*δ*_C_ 51.7), from H_3_-13 to C-12 (*δ*_C_ 25.6), from H_2_-14 to C-7 and C-8 defined the ring B fragment and planar structure of **2** ultimately (Fig. 2).

**Fig. 4:**
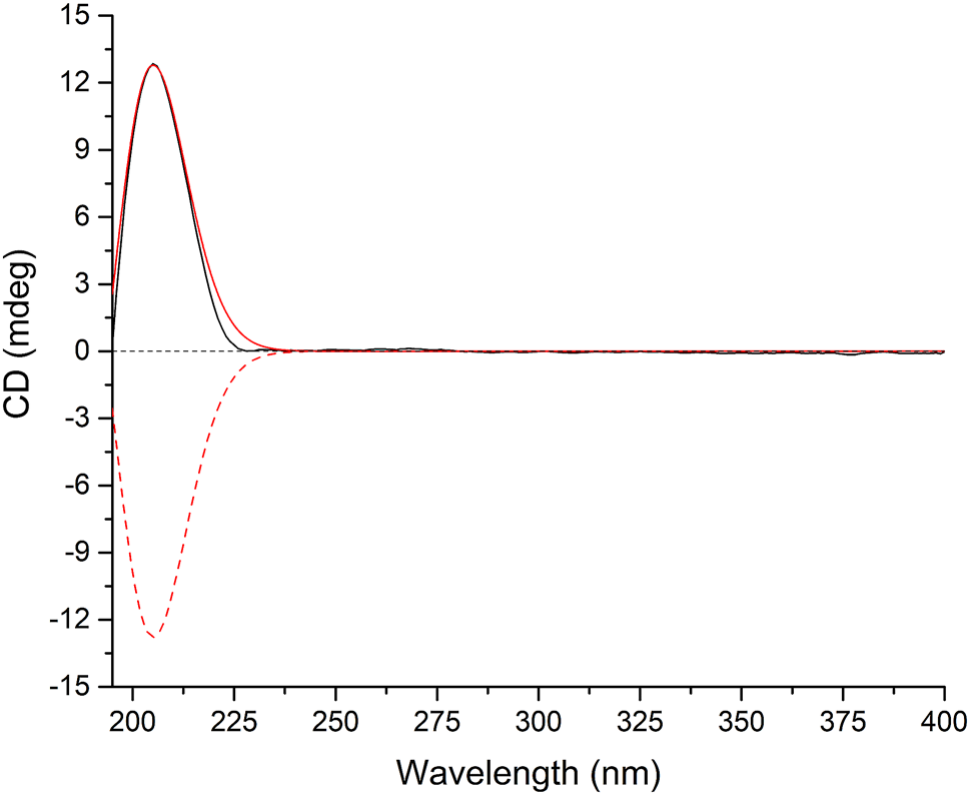
Experimental ECD spectrum of **2** (black); Calculated ECD spectra of (1*R*,2*R*,4*S*,5*S*)-**2** (shift = 12.5 nm, red) and (1*S*,2*S*,4*R*,5*R*)-**2** (shift = 12.5 nm, red dash)

As for the configuration of **2**, randomly assigning H-5 as *β*-oriented, the observed correlations of H_2_-10/H_3_-11/H-5 in the ROESY spectrum, together with the coupling constant (*J* = 4.4 Hz) between H-1 and H-2 implied that both CH_2_OH-10 and CH_3_-11 adopted *β*-orientation, while the analysis of coupling constant (*J* = 10.8 Hz) between H-4 and H-5 suggested that H-4 was *α*-oriented (Fig. 2). Then, (1R*,2R*,4S*,5S*)-**2** was subjected to quantum chemical calculation of NMR chemical shifts and spin–spin coupling constants at mPW1PW91-SCRF/6–31 + G(d,p)//B3LYP-D3BJ-SCRF/6-31G(d) and B972-SCRF/pcJ-1//B3LYP-D3BJ-SCRF/6-31G(d) level of theory (both with methanol as solvent and SMD solvent model), respectively, and the calculated results matched their experimental counterparts very well (Tables 3 and S3), which verified the established planar structure and relative configuration of **2**. Moreover, TDDFT ECD calculation of (1*R*,2*R*,4*S*,5*S*)-**2** was run at CAM-B3LYP-SCRF/def2-SVP//B3LYP-D3BJ-SCRF/6-31G(d) level of theory in MeOH with SMD solvent model, and the obtained curve, which is entirely consistent with its experimental counterpart, supported the absolute configuration of **2** to be 1*R*,2*R*,4*S*, and 5*S* (Fig. 4). Therefore, compound **2** was confirmed to possess 15-nor-botryane skeleton and named as arthrinin F.

**Table 3:** Analyses of the NMR computation results of (1R*,2R*,4S*,5S*)-**2** and (6R*,8S*)-**3**

Parameters	R^2^	MAE (ppm)	CMAE (ppm)
(1R*,2R*,4S*,5S*)-**2**			
^13^C	0.9992	1.3	0.8
^1^H	0.9938	0.1	0.06
(6R*,8S*)-**3**			
^13^C	0.9989	1.5	1.4
^1^H	0.9948	0.21	0.13

Compound **3** was isolated as a colorless oil. The [M+Na]^+^ peak at *m/z* 285.1100 (calcd for 285.1097) existing in the positive HRESIMS spectrum revealed the molecular formula of **3** to be C_15_H_18_O_4_, which implied seven indices of hydrogen deficiency. Its IR spectrum indicated the presence of hydroxyl (3419 cm^–1^), methyl (2932 and 2869 cm^–1^), phenyl (1607 and 1467 cm^–1^) and ester (1743 and 1040 cm^–1^) groups. The ^1^H NMR spectrum gave two characteristic aromatic protons at *δ*_H_ 7.43 (d, *J* = 7.9 Hz), 7.72 (d, *J* = 7.9 Hz) and two singlet methyl groups (*δ*_H_ 1.37, 1.34) (Table 1). The ^13^C NMR and DEPT spectra displayed 15 carbon signals, including one ester carbonyl carbon, six aromatic carbons, two *sp*^3^ quaternary carbons, four *sp*^3^ methylenes and two methyls (Table 2). The above data suggested that the structure of **3** resembled to that of dehydrobotrylactone with a botryane scaffold [23].

The ^1^H-^1^H COSY correlation between H-3 and H-4, along with the HMBC correlations from H-3 to C-1 (*δ*_C_ 145.2), C-5 (*δ*_C_ 157.8) and C-11 (*δ*_C_ 173.9), from H-4 to C-2 (*δ*_C_ 126.0) and C-9 (*δ*_C_ 144.7), from H_2_-10 to C-1, C-2 and C-11 established the structure of the butyrolactone motif (ring C), and its connection to a tetra-substituted benzene ring (ring A). Moreover, the HMBC cross peaks from H-4 to C-6 (*δ*_C_ 50.1), from H_2_-7 to C-13 (*δ*_C_ 27.2) and C-15 (*δ*_C_ 70.8), from H_2_-12 to C-5 and C-6, from H_3_-13 to C-12 (*δ*_C_ 71.3), from H_3_-14 to C-8 (*δ*_C_ 50.2) and C-9, from H_2_-15 to C-14 (*δ*_C_ 24.9) determined the existence of a cyclopentane fragment (ring B) and its linkage with ring A. As a result, the planar structure of compound **3** was elucidated as shown in Fig. 2.

**Fig. 5:**
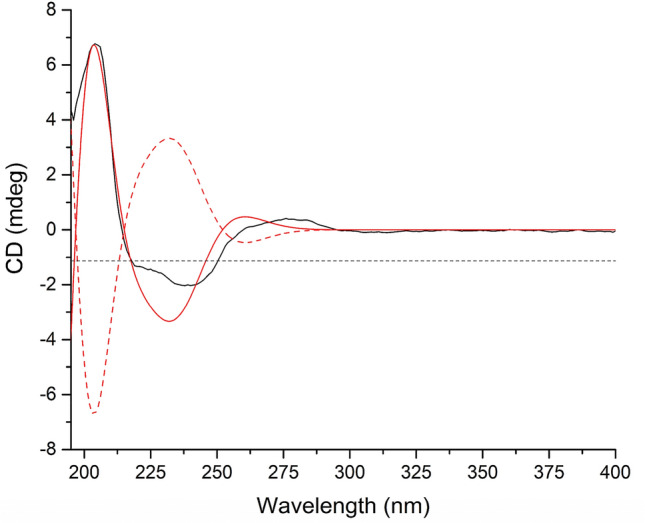
Experimental ECD spectrum of **3** (black); Calculated ECD spectra of (6*R*,8*S*)-**3** (shift = 12 nm, red) and (6*S*,8*R*)-**3** (shift = 12 nm, red dash)

As for the stereochemistry of **3**, randomly assigning H-7a as *α*-oriented, according to the ROESY correlations observed from H-7*α* to both H_3_-13 and H_3_-14, as well as from H-7*β* to both H_2_-12 and H_2_-15, it can be concluded that both CH_3_-13 and CH_3_-14 adopted *α*-orientation. Then, the NMR chemical shifts of (6R*,8S*)-**3** were calculated at mPW1PW91-SCRF/6–31 + G(d,p)//B3LYP-D3BJ-SCRF/6-31G(d) level of theory with methanol as solvent and SMD solvent model, the predicted chemical shifts matched their experimental counterparts very well (Table 3), which supported the above deduction concerning the relative configuration of **3**. Furthermore, the theoretical ECD spectrum of (6*R*,8*S*)-**3** was obtained at CAM-B3LYP-SCRF/def2-SVP//B3LYP-D3BJ-SCRF/6-31G(d) level of theory in MeOH with SMD solvent model, and the calculated curve matched the experimental one very well and thus supported the absolute configuration of **3** to be 6*R*,8*S*. (Fig. 5). Compound **3** was given the trivial name arthrinin G.

Additionally, compounds **1**–**3** were evaluated for their cytotoxicity against five human cancer cell lines (HL-60, A549, SMMC-7721, MCF-7, SW480), with *cis*-platin and paclitaxel as positive controls, however, no compounds showed activity against the tested cell lines (Fig. S2).

## Experimental

### General Experimental Procedures

Optical rotations were measured with a JASCO P-1020 polarimeter. UV spectra were obtained using a Shimadzu UV-2401 PC spectrophotometer. A Tensor 27 spectrophotometer was used for scanning IR spectroscopy with KBr pellets. 1D and 2D NMR spectra were recorded on Bruker DRX-600 and 500 spectrometers with TMS as internal standard. Chemical shifts (*δ*) are expressed in parts per million (ppm) with reference to the solvent signals. HRESIMS was performed on an API QSTAR spectrometer. Semipreparative HPLC was performed on an Agilent 1200 liquid chromatograph with a Zorbax SB-C18 (9.4 mm × 250 mm) column. LC–MS/MS was performed on Agilent 6530 Accurate-Mass Q-TOF spectrometer coupled to an Agilent 1290 LC system with Zorbax SB-C18 (9.4 mm × 250 mm) column. Column chromatography was performed with silica gel (100–200 mesh, Qingdao Marine Chemical, Inc., Qingdao, People’s Republic of China). Fractions were monitored by TLC, and spots were visualized by heating silica gel plates sprayed with 10% H_2_SO_4_ in EtOH.

### Fungal Material, Identification and Fermentation

The fungal strain of *Arthrinium* sp. HS66 was isolated from the fresh stems of *Isodon xerophilus* that was collected from Kunming Botanical Garden, Kunming City, Yunnan Province, People’s Republic of China, in August 2018. The isolate was identified based on sequence (GenBank Accession No. MT355097) analysis of the ITS region of the rDNA. The fungal strain was cultured on slants of potato dextrose agar at 28 °C for 7 days. Agar plugs were cut into small pieces (about 0.5 × 0.5 × 0.5 cm^3^) under aseptic conditions, and 15 pieces were used to inoculate three Erlenmeyer flasks (500 mL), each containing 200 mL of media (0.4% glucose, 1% malt extract, and 0.4% yeast extract); the final pH of the media was adjusted to 7.0, and the flasks were sterilized by autoclave. Three flasks of the inoculated media were incubated at 28 °C on a rotary shaker at 170 rpm for 7 days to prepare the seed culture. The detailed lager fermentation procedure was as following: Fermentation was carried out on solid rice medium in 125 Fernbach flasks (500 mL, 90 mL distilled water was added to 80 g rice and kept overnight before autoclaving). Each flask was inoculated with 5.0 mL of the spore inoculum and incubated for 30 days at 28 °C in a static incubator.

### Cytotoxicity Assay

Five human cancer cell lines, human myeloid leukemia HL-60, lung cancer A-549 cells, hepatocellular carcinoma SMMC-7721, breast cancer MCF-7, and colon cancer SW480, were purchased from the Shanghai Institute of Biochemistry and Cell Biology, Chinese Academy of Sciences (Shanghai, China). Cells were cultured according to the manufacturer’ recommendations. All mediums were supplemented with 10% fetal bovine serum (FBS), 100 units/ml penicillin G sodium and 100 μg/ml streptomycin (HyClone). All the cells were incubated at 37 ℃, 5% CO_2_ in a humidified atmosphere. Cytotoxicity of compounds was determined by MTS method. Briefly, 5 × 10^3^ cells were plated in 96-well plates 12 h before treatment and continuously exposed to test compounds for 48 h. Then MTS (Promega) was added to each well. The samples were incubated at 37 ℃ for 1–4 h and the optical density (OD) was measured at 490 nm using a microplate reader (Bio-Rad Laboratories). The IC_50_ values were calculated by Reed and Muench’s method [24].

### Extraction and Isolation of Compounds 1–3

The culture medium was overlaid and extracted with MeOH by maceration. With filtration and concentration, the resultant extract was partitioned with EtOAc. Then the solvent was evaporated in vacuo to afford a crude extract (130 g). The extraction was subjected to column chromatography on silica gel with a CHCl_3_/Me_2_CO gradient system (1:0, 9:1, 8:2, 7:3, 6:4, 1;1, 0:1) to yield seven fractions, A-G. Fraction C (CHCl_3_/Me_2_CO 8:2, 9 g) was chromatographed on a RP-18 column with a methanol/ H_2_O gradient system (from 30:70 to 100:0) to afford fractions C1-C8. Fraction C2 (methanol/ H_2_O, 40:60, 1.4 g) was subjected to chromatography over silica gel (chloroform/ Me_2_CO, from 80:1 to 0:1) to yield subfractions C2/1–11, subfraction C2-9 was purified by semipreparative HPLC (3 ml/min, detector UV *λ*_max_ = 195 nm, MeCN/H_2_O 22.5:77.5) to yield **1** (3.1 mg, t_R_ = 18.7 min) and **2** (1.8 mg, t_R_ = 20.2 min), subfraction C2-10 was purified by semipreparative HPLC (3 ml/min, detector UV *λ*_max_ = 195 nm, MeCN/H_2_O 40:60) to yield **3** (3.6 mg, t_R_ = 25.8 min).

### Physical Constants and Spectroscopic Data of Compounds 1–3

Arthrinin E (**1**): initially obtained as a colorless oil, by using slow evaporation of methanol in a closed tube, the colorless square crystals were obtained eventually; mp: 135–140 °C; [*α*]_D_^22.2^: + 66.6 (MeOH, *c* 0.100); ECD (MeOH) *λ*_max_ (Δ*ε*): 220 (0.02) nm; UV (MeOH) *λ*_max_ (log *ε*): 204 (3.03) nm; IR (*ν*_max_): 3374, 2927, 2869, 1630, 1607, 1460, 1384, 1366, 1093, 1057, 1026, 1010, 986, 971 cm^–1^. HRESIMS at *m/z* 277.1775 ([M+Na]^+^, calcd for 277.1774). ^1^H and ^13^C NMR data, see Tables 1 and 3.

Arthrinin F (**2**): obtained as a colorless oil; [*α*]_D_^22.4^: + 107.00 (MeOH, *c* 0.060); ECD (MeOH) *λ*_max_ (Δ*ε*): 205 (0.68) nm; UV (MeOH) *λ*_max_ (log *ε*): 201 (3.14) nm; IR (*ν*_max_): 3383, 2955, 2927, 2890, 2871, 1631, 1609, 1465, 1454, 1384, 1361, 1082, 1026, 994, 978 cm^–1^. HRESIMS at *m/z* 263.1618 ([M + Na]^+^, calcd for 263.1618). ^1^H and ^13^C NMR data, see Tables 1 and 3.

Arthrinin G (**3**): isolated as a colorless oil; [*α*]_D_^22.8^: + 12.55 (MeOH, *c* 0.110); ECD (MeOH) *λ*_max_ (Δ*ε*): 204 (0.21), 240 (– 0.06), 276 (0.01) nm; UV (MeOH) *λ*_max_ (log *ε*): 208 (3.22), 241 (2.74), 283 (1.98) nm; IR (*ν*_max_): 3419, 2957, 2932, 2869, 1743, 1607, 1467, 1384, 1367, 1040, 1017, 744 cm^–1^. HRESIMS at *m/z* 285.1100 ([M+Na]^+^, calcd for 285.1097). ^1^H and ^13^C NMR data, see Tables 1 and 3.

Crystallographic data for the structures of arthrinin E (**1**, deposition number CCDC 1997674) has been deposited in the Cambridge Crystallographic Data Centre database. Copies of the data can be obtained free of charge from the CCDC at www.ccdc.cam.ac.uk.

*Crystal data for***1**: C_15_H_26_O_3_, *M* = 254.36, *a* = 8.8861(2) Å, *b* = 8.8861(2) Å, *c* = 31.7909(6) Å, *α* = 90°, *β* = 90°, *γ* = 120°, *V* = 2173.98(11) Å^3^, *T* = 100.(2) K, space group *P*3221, *Z* = 6, *μ*(Cu K*α*) = 0.629 mm^–1^, 29,180 reflections measured, 2838 independent reflections (*R*_*int*_ = 0.0291). The final *R*_*1*_ values were 0.0351 (*I* > 2*σ*(*I*)). The final *wR*(*F*^2^) values were 0.0969 (*I* > 2*σ*(*I*)). The final *R*_*1*_ values were 0.0353 (all data). The final *wR*(*F*^2^) values were 0.0970 (all data). The goodness of fit on *F*^2^ was 1.142. Flack parameter = 0.07(3).

### Computational Method

Conformational searching of (1R*,2R*,4S*,5S*)-**2** and (6R*,8S*)-**3** were undertaken with the CREST code (version 2.8) using the default iMTD-GC procedure [25]. The first 20 conformers of (1R*,2R*,4S*,5S*)-**2** and (6R*,8S*)-**3** were subjected to DFT geometry optimization at B3LYP-D3BJ-SCRF/6-31G(d) level of theory (with MeOH as solvent and SMD solvent model). Frequency analyses of all optimized conformers were undertaken at the same level of theory to ensure that no imaginary frequency exists. Then, thermal correction to Gibbs free energies obtained by frequency analyses were added to the electronic energies obtained at B3LYP-D3BJ-SCRF/6–311 + G(d,p) level of theory (with MeOH as solvent and SMD solvent model) to get the Gibbs free energies of each conformer. Subsequently, Room-temperature (298.15 K) equilibrium populations were calculated according to Boltzmann distribution law:

$${p}_{i}= \frac{{n}_{i}}{{\sum }_{j}{n}_{j}}= \frac{{e}^{-\Delta {G}_{i}/RT}}{{\sum }_{j}{e}^{-\Delta {G}_{j}/RT}}$$

where *P*_*i*_ is the population of the *ith* conformer; *n*_*i*_ the number of molecules in *ith* conformer; *ΔG* is the relative Gibbs free energy (kcal/mol); *T* is room temperature (298.15 K) here; *R* is the ideal gas constant (0.0019858995). Those conformers with a population of over 2% were subjected to subsequent NMR and ECD calculations.

NMR shielding constants were calculated with the GIAO method at mPW1PW91-SCRF/6–31 + G(d,p) level (with MeOH and SMD solvent model). The obtained shielding constants were converted into chemical shifts by referencing to TMS at 0 ppm (*δ*_cal_ = *σ*_TMS_ – *σ*_cal_), where the *σ*_TMS_ was the shielding constant of TMS calculated at the same level of theory. The parameters *a* and *b* of the linear regression *δ*_cal_ = *aδ*_exp_ + *b*; the correlation coefficient, *R*^2^; the mean absolute error (MAE) defined as Σ_n_|*δ*_cal_ – *δ*_exp_|/*n*; the corrected mean absolute error (CMAE) defined as Σ_n_|*δ*_corr_ – *δ*_exp_|/*n*, where *δ*_corr_ = (*δ*_cal_ – *b*)/*a* were calculated [26, 27]. Calculation of coupling constants were run at B972/pcJ-1 level of theory (with MeOH as solvent and SMD solvent model) [28].

TDDFT ECD calculations were run at CAM-B3LYP/def2-SVP level of theory (with MeOH as solvent and SMD solvent model) [29]. For each conformer, 30 excited states were calculated. The calculated ECD curves were generated using the Multiwfn software (version 3.7) [30].

The geometry optimization, single-point energy calculation, NMR shielding constant calculation, coupling constant calculation, and TDDFT ECD calculation were all completed in Gaussian 09 program [31].

## Electronic supplementary material

Below is the link to the electronic supplementary material.

Electronic supplementary material 1 (PDF 3960 kb)
