# Supplementary material for: Arthrinins E–G, Three Botryane Sesquiterpenoids from the Plant Endophytic Fungus Arthrinium sp. HS66
Source: Nat Prod Bioprospect. 2020 Jul 12;10(4):201–7. doi: 10.1007/s13659-020-00248-y (PMC7367987; doi:10.1007/s13659-020-00248-y)
Supplement: Supplementary file 1 — Electronic supplementary material 1 (PDF 3960 kb) [file 13659_2020_248_MOESM1_ESM.pdf]

Supporting Information for

Arthrinins E–G, three botryane sesquiterpenoids from the plant endophytic fungus *Arthrinium* sp. HS66

Xiao-Zheng Su,[a,b,c] Jian-Wei Tang,[a,b] Kun Hu,[a,b] Xiao-Nian Li,[a,b] Han-Dong Sun,[a,b] Pema-Tenzin Puno*[a,b]

[a] State Key Laboratory of Phytochemistry and Plant Resources in West China, Kunming Institute of Botany, Chinese Academy of Sciences, Kunming 650201, People´s Republic of China

[b] Yunnan Key Laboratory of Natural Medicinal Chemistry, Kunming Institute of Botany, Chinese Academy of Sciences, Kunming 650201, People´s Republic of China

[c] University of Chinese Academy of Sciences, Beijing 100049, People´s Republic of China

* Corresponding author Tel.: (86) 871-65223616
E-mail: punopematenzin@mail.kib.ac.cn.

Contents of Supporting Information

**Contents**	**Pages**
Experimental details	3
Figure S1. X-ray structure of **1**	3
Table S20-27. X-ray crystal detailed data of **1**	48-52
Figure S2. Cytotoxicity results of **1**–**3**	3
Table S1-19. Computational data of **2** and **3**	3-6, 32-47
Figure S3-6. Computational data of **2** and **3**	5-7
Figure S7-39. 1D, 2D NMR, HRESIMS, ESI, IR, UV and CD spectra of **1**–**3**	8-31

Experimental details

1. *X-ray Crystal Structure Analysis*

The intensity data for arthrinin E (**1**) were collected on a Bruker APEX DUO diffractometer using graphite-monochromated Cu Kα radiation. The structure of the compound was solved by direct methods (SHELXS97), expanded using difference Fourier techniques, and refined by the program and full-matrix least-squares calculations. The non-hydrogen atoms were refined anisotropically, and hydrogen atoms were fixed at calculated positions. Detailed data were provided in Tables S20-27 in the Supporting Information.

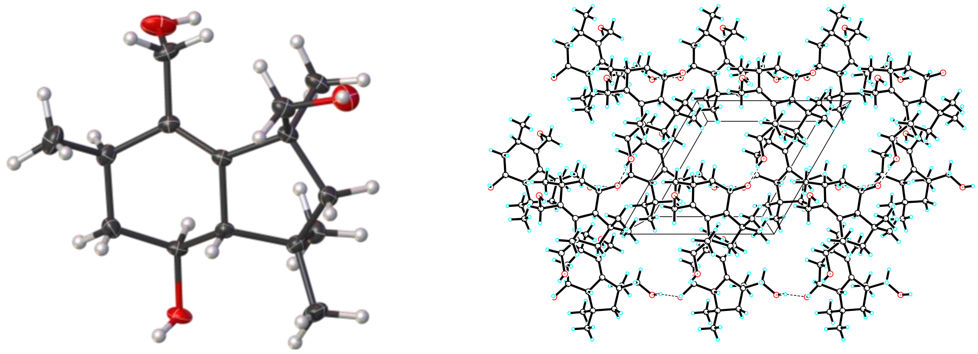

Figure S1. X-ray crystal structure of arthrinin E (**1**).

2. *Cytotoxicity results*

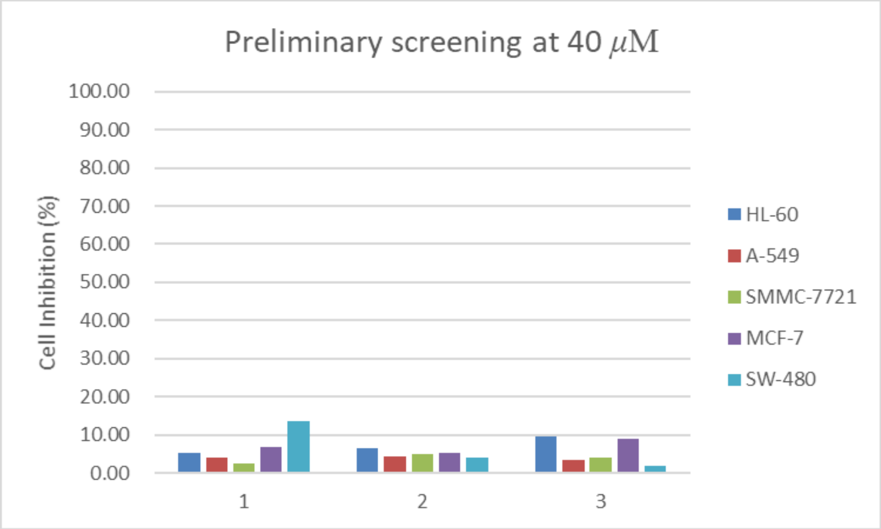

Figure S2. Effects of compounds **1**–**3** on the growth of various cell lines were analyzed by MTS assay.

3. *Computational data of compounds 2 and 3*

Table S1. Experimental and calculated ^{13}C-NMR chemical shifts of (1*R**, 2*R**, 4*S**, 5*S**)-**2**

No.	$\delta_{exptl.}$	$\delta_{calcd.}$
1	43.5	44.8
2	33.7	36.0

3	40.8	41.4
4	71.9	74.3
5	60.9	60.9
6	39.7	42.7
7	51.7	51.4
8	136.9	139.3
9	139.8	141.8
10	59.9	61.1
11	19.4	19.9
12	31.6	30.7
13	25.6	25.6
14	59.2	60.4

Table S2. Experimental and calculated ^{1}H-NMR chemical shifts of (1*R**, 2*R**, 4*S**, 5*S**)-**2**

No.	$\delta_{exptl.}$	$\delta_{calcd.}$
1	2.6	2.56
2	1.67	1.6
3α	1.67	1.44
3β	1.31	1.18
4	3.57	3.66
5	2.3	2.37
7α	2.16	2.08
7β	2.37	2.53
10	3.45	3.46
10	3.69	3.66
11	1.03	0.85
12	1.23	1.07
13	1.07	0.89
14	4.05	4.03

Table S3. Experimental and calculated spin-spin coupling constants of (1*R**, 2*R**, 4*S**, 5*S**)-**2**

^3J	**H-1/H-10a**	**H-1/H-10b**	**H-1/H-2**	**H-4/H-3α**	**H-4/H-3β**	**H-4/H-5**
**Calculated**	4.3	10.6	5.2	4.3	10.2	9.7
**Experimental**	4.4	10.2	4.4	4.5	10.8	10.8
**Absolute error**	0.13	0.38	0.8	0.17	0.64	1.14

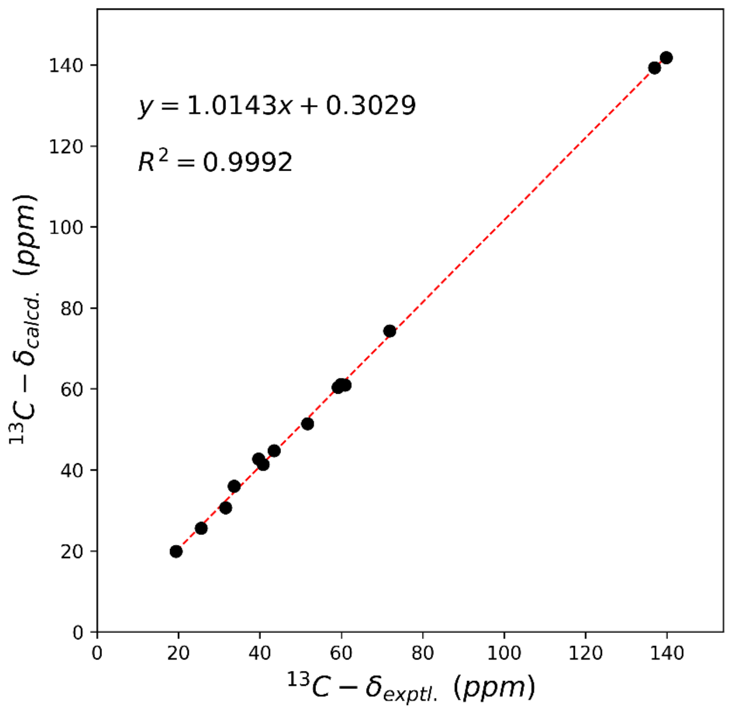

Figure S3. Linear regression analysis between experimental and calculated ^{13}C NMR chemical shifts of (1*R**, 2*R**, 4*S**, 5*S**)-**2**.

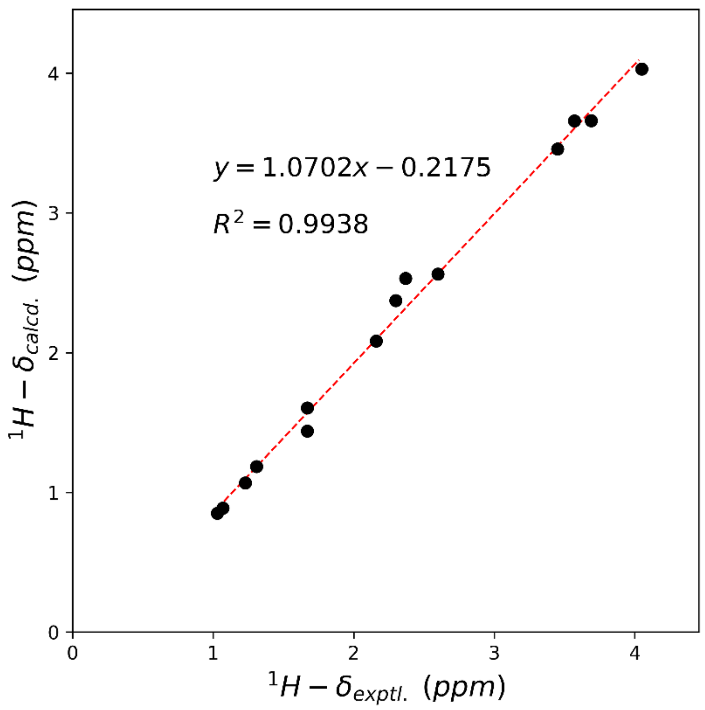

Figure S4. Linear regression analysis between experimental and calculated ^{1}H NMR chemical shifts of (1*R**, 2*R**, 4*S**, 5*S**)-**2**.

Table S4. Experimental and calculated ^{13}C-NMR chemical shifts of (6*R**, 8*S**)-**3**

No.	$\delta_{exptl.}$	$\delta_{calcd.}$
1	145.2	144.9
2	126.0	122.7
3	125.4	124.7
4	126.1	123.7
5	157.8	158.0
6	50.1	54.3
7	47.8	47.6
8	50.2	53.7
9	144.7	142.8
10	71.1	72.2
11	173.9	173.1
12	71.3	73.2
13	27.1	28.0
14	24.9	25.4
15	70.8	70.4

Table S5. Experimental and calculated ^{1}H-NMR chemical shifts of (6*R**, 8*S**)-**3**

No.	$\delta_{exptl.}$	$\delta_{calcd.}$
3	7.72	8.21
4	7.43	7.78
7α	1.7	1.72
7β	2.36	2.79
10	5.47	5.55
10	5.55	5.65
12	3.58	3.61
12	3.61	3.95
13	1.34	1.18
14	1.37	1.17
15	3.64	3.78

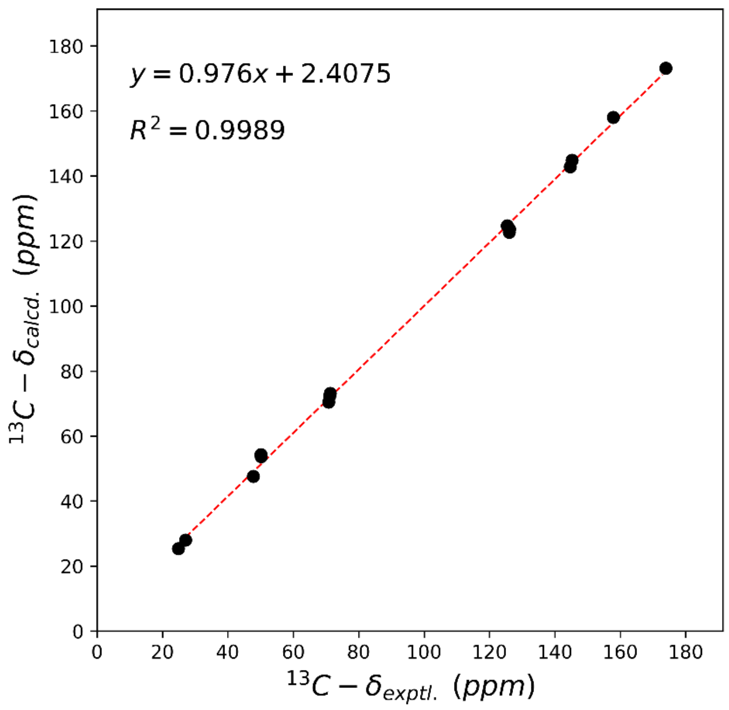

Figure S5. Linear regression analysis between experimental and calculated ^{13}C NMR chemical shifts of (6*R**, 8*S**)-**3**.

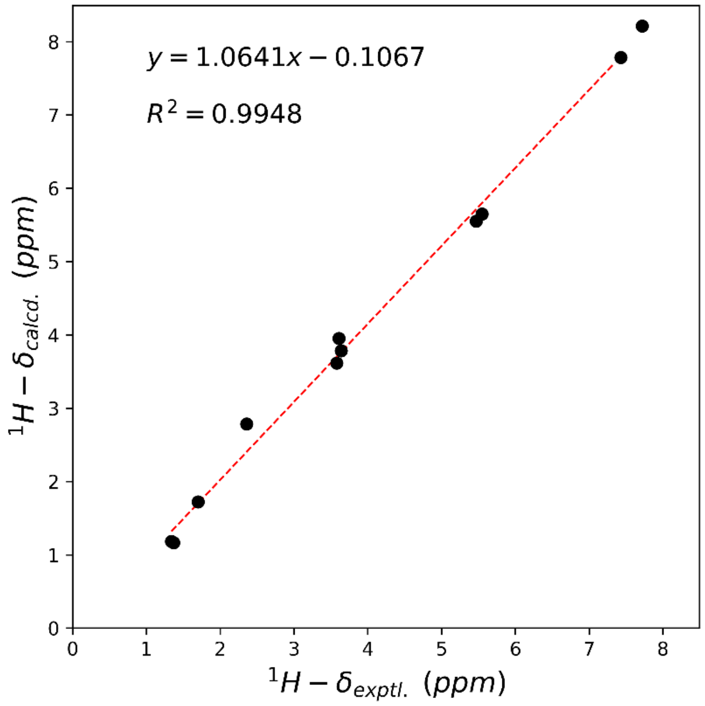

Figure S6. Linear regression analysis between experimental and calculated ^{1}H NMR chemical shifts of (6*R**, 8*S**)-**3**.

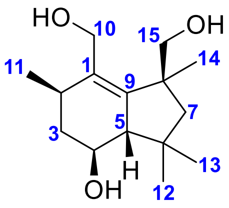

Arthrinin E

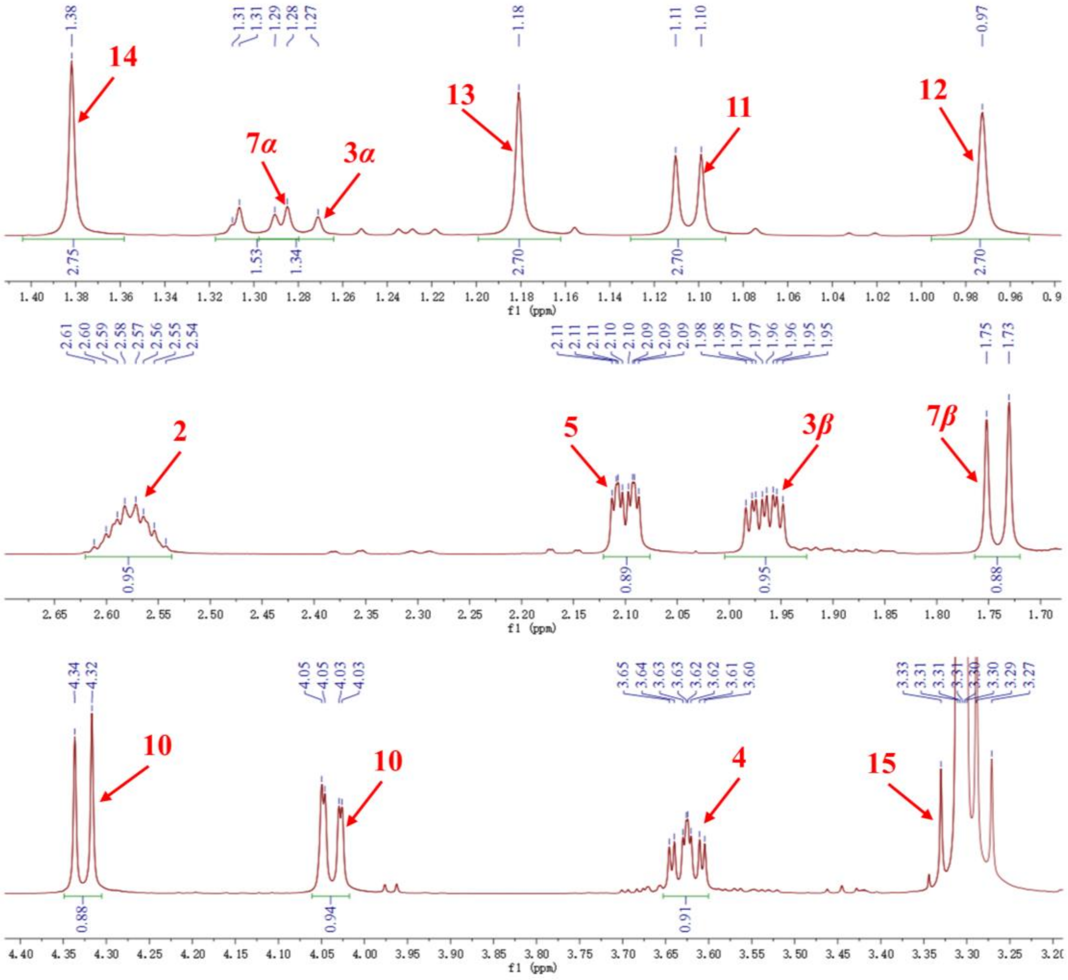

Figure S7. ^{1}H NMR spectrum of arthrinin E (**1**) (CD_3OD).

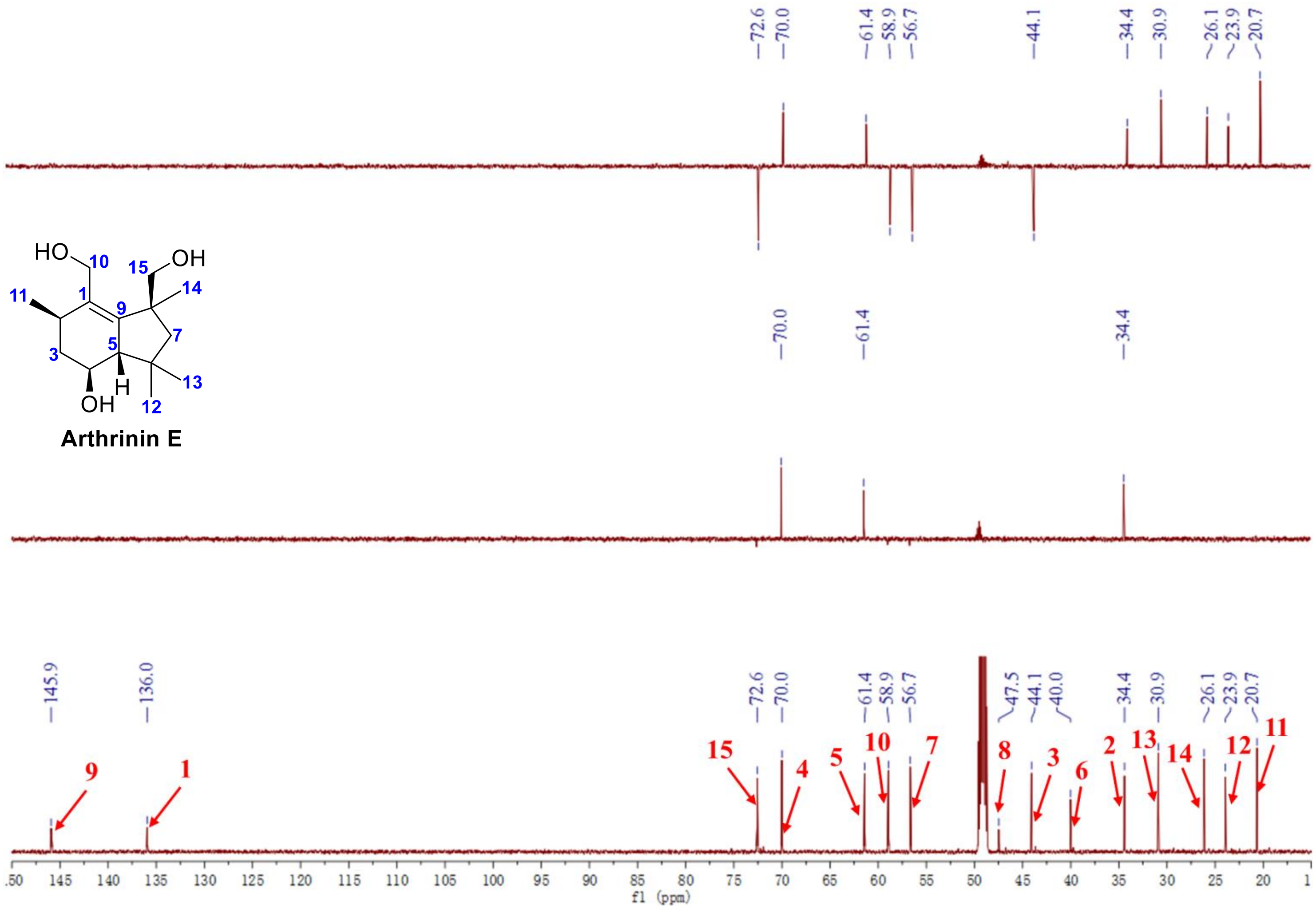

Figure S8. ^{13}C NMR and DEPT spectrum of arthrinin E (**1**) (CD_3OD).

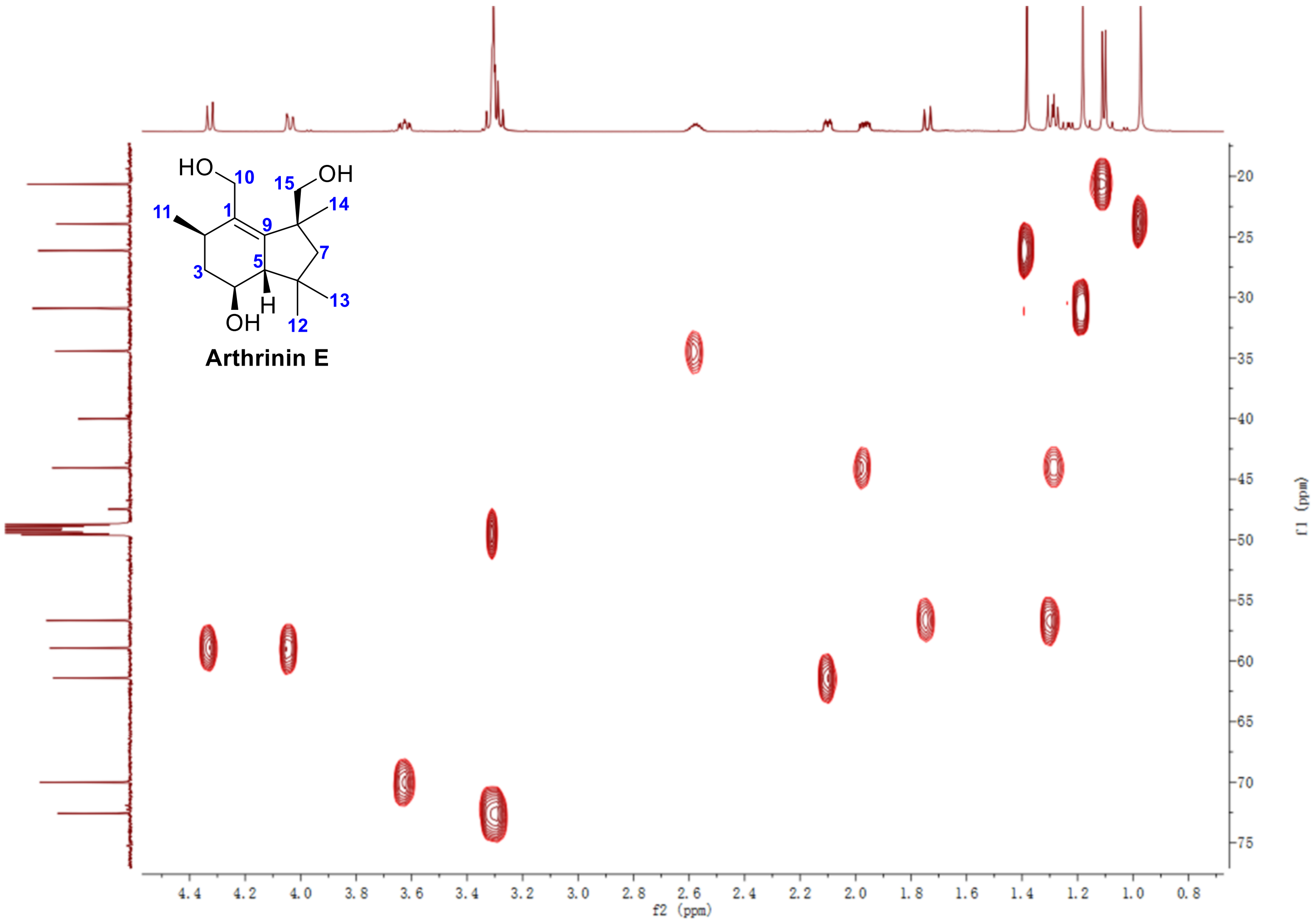

Figure S9. HSQC spectrum of arthrinin E (**1**) (CD_3OD).

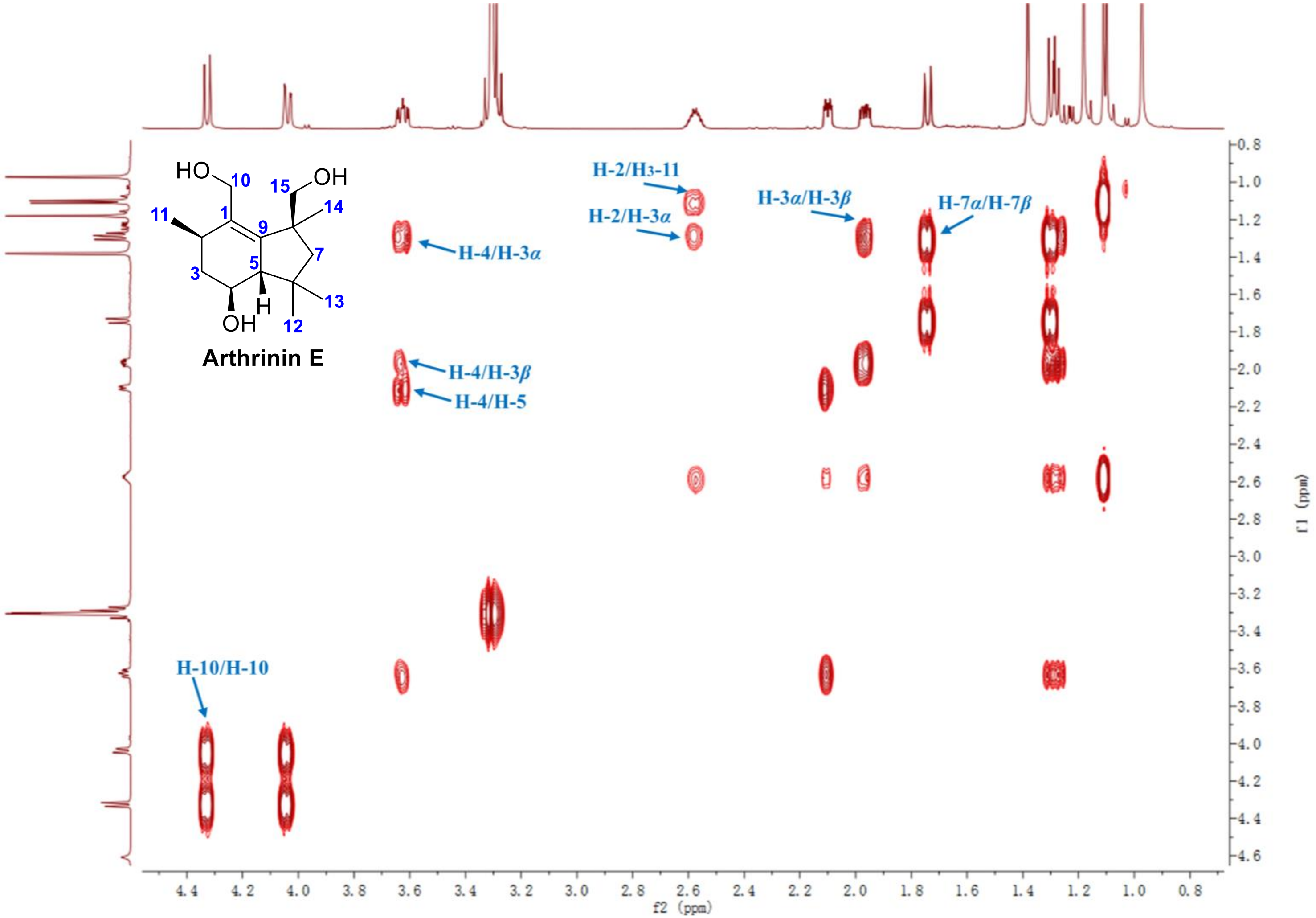

Figure S10. ^{1}H-^{1}H COSY spectrum of arthrinin E (**1**) (CD_3OD) and the selected cross-peaks (blue arrow and text).

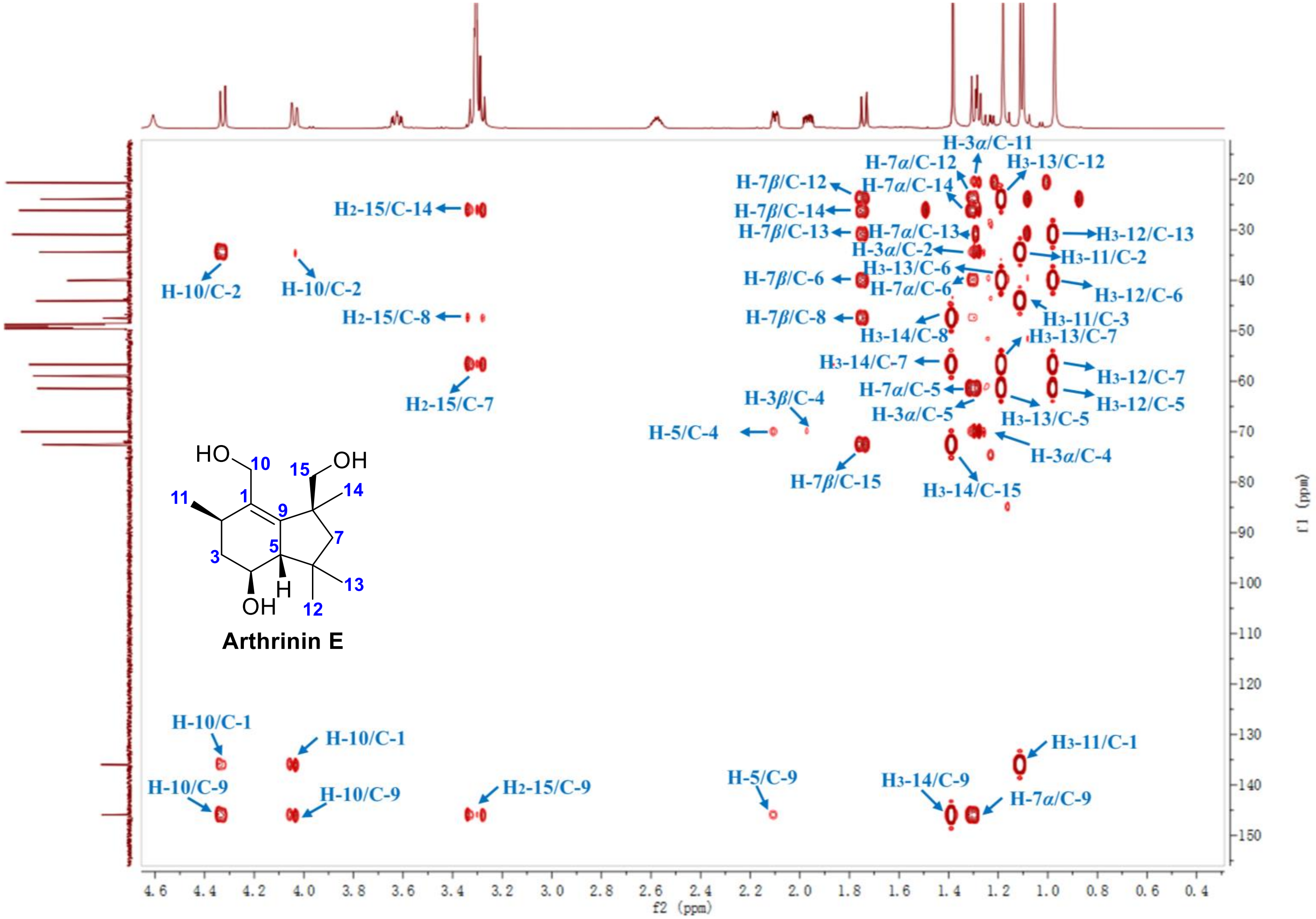

Figure S11. HMBC spectrum of arthrinin E (**1**) (CD_3OD) and the selected correlations (blue arrow and text).

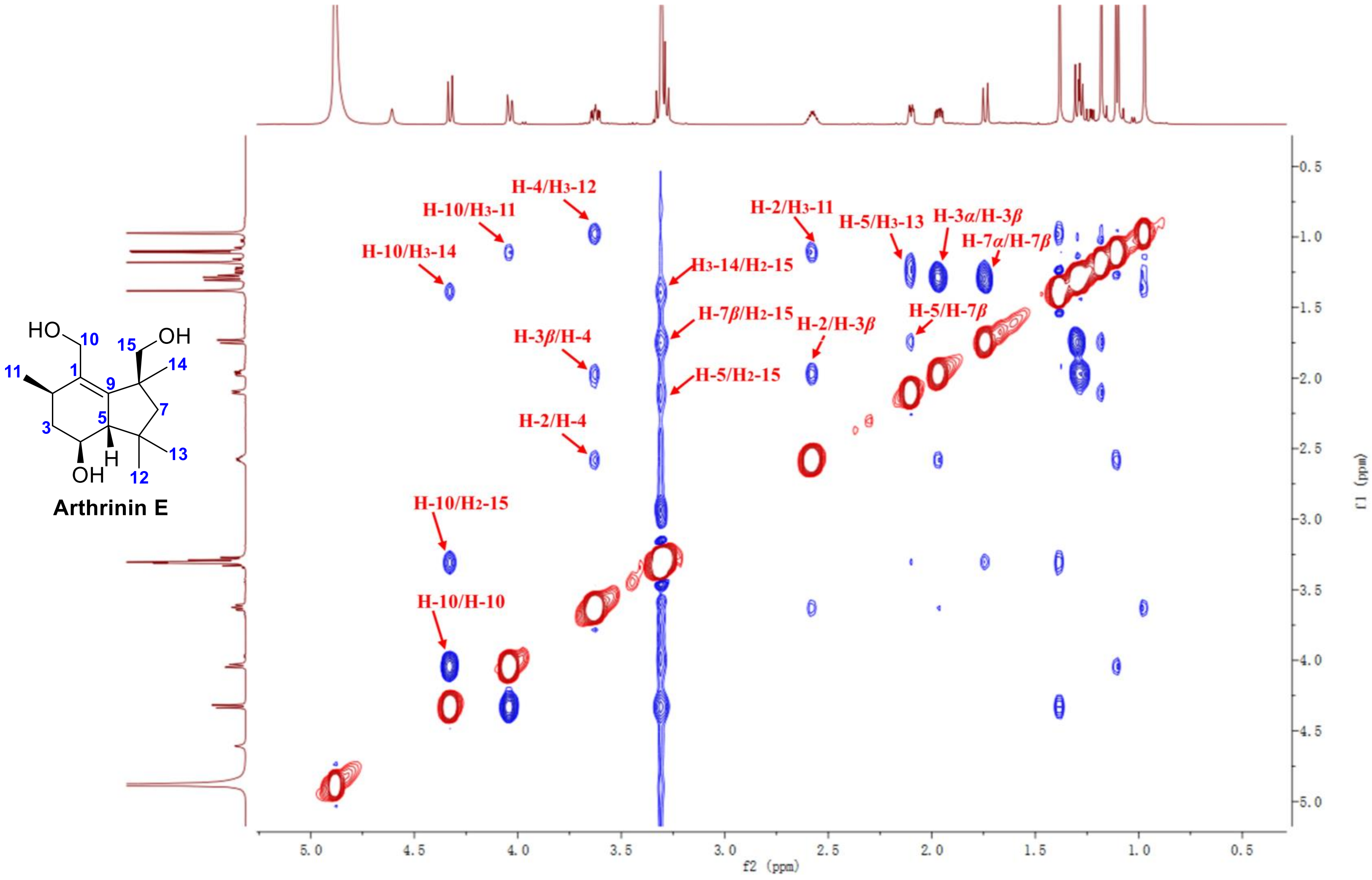

Figure S12. ROESY spectrum of arthrinin E (**1**) (CD_3OD) and the selected correlations (red arrow and text).

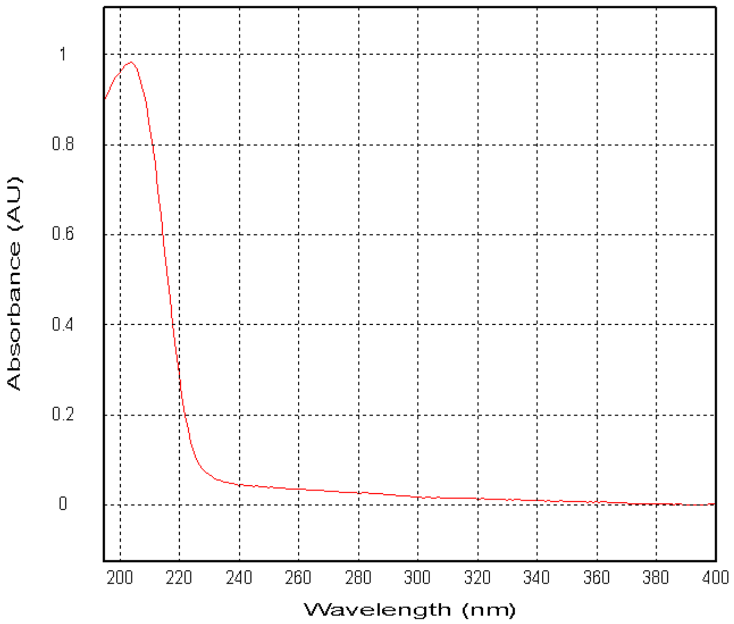

Figure S13. UV spectrum of arthrinin E (**1**).

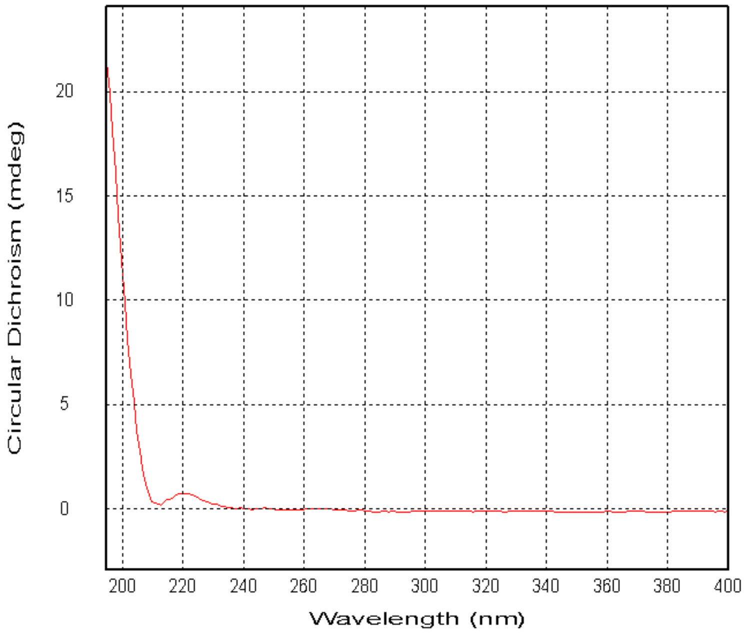

Figure S14. CD spectrum of arthrinin E (**1**).

n	Average	Std.Dev.	% RSD	Maximum	Minimum
5	66.60	0.55	0.82	67.00	66.00

S.No	Sample ID	Time	Result	Scale	OR °Arc	WLG.nm	Lg.mm	Conc.g/100ml	Temp.
1	SHSJ10a	05:13:02 PM	67.00	SR	0.067	589	100.00	0.100	22.2
2	SHSJ10a	05:13:08 PM	66.00	SR	0.066	589	100.00	0.100	22.2
3	SHSJ10a	05:13:13 PM	66.00	SR	0.066	589	100.00	0.100	22.2
4	SHSJ10a	05:13:19 PM	67.00	SR	0.067	589	100.00	0.100	22.2
5	SHSJ10a	05:13:25 PM	67.00	SR	0.067	589	100.00	0.100	22.2

Figure S15. OR report of arthrinin E (**1**).

Sample Group		Info.
Acquisition SW Version	6200 series TOF/6500 series Q-TOF B.05.01 (B5125.2)	

User Spectra

Fragmentor Voltage	Collision Energy	Ionization Mode
200	0	ESI

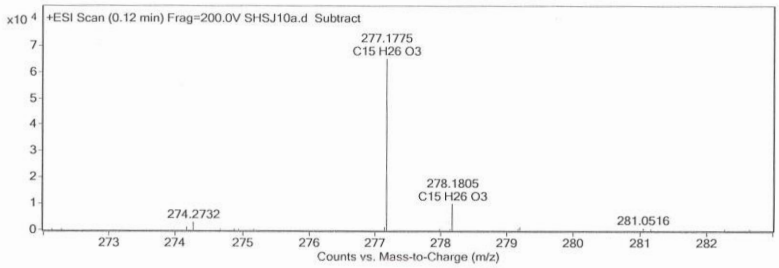

Peak List

m/z	z	Abund	Formula	Ion
189.1637	1	21920.52		
219.1745		5139.8		
277.1775	1	65107.77	C15 H26 O3	(M+Na)+
278.1805	1	10016.14	C15 H26 O3	(M+Na)+
293.1725	1	6303.07		
353.2663		4566.59		
413.2665	1	6455.12		
437.1936	1	13273.71		
610.1848	1	7861.26		
659.2866	1	7321.34		

Formula Calculator Element Limits

Element	Min	Max
C	3	60
H	0	120
O	0	30

Formula Calculator Results

Formula	CalculatedMass	CalculatedMz	Mz	Diff. (mDa)	Diff. (ppm)	DBE
C15 H26 O3	254.1882	277.1774	277.1775	-0.10	-0.36	3.0000

Figure S16. HRESIMS spectrum of arthrinin E (**1**).

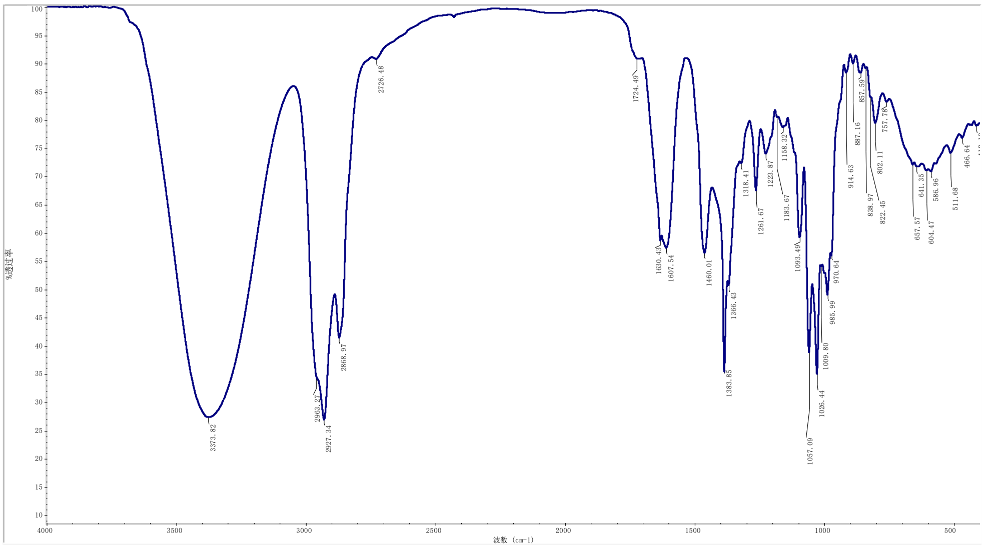

Figure S17. IR spectrum of arthrinin E (**1**).

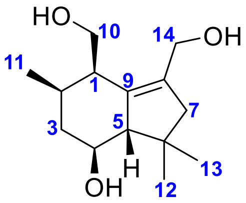

Arthrinin F

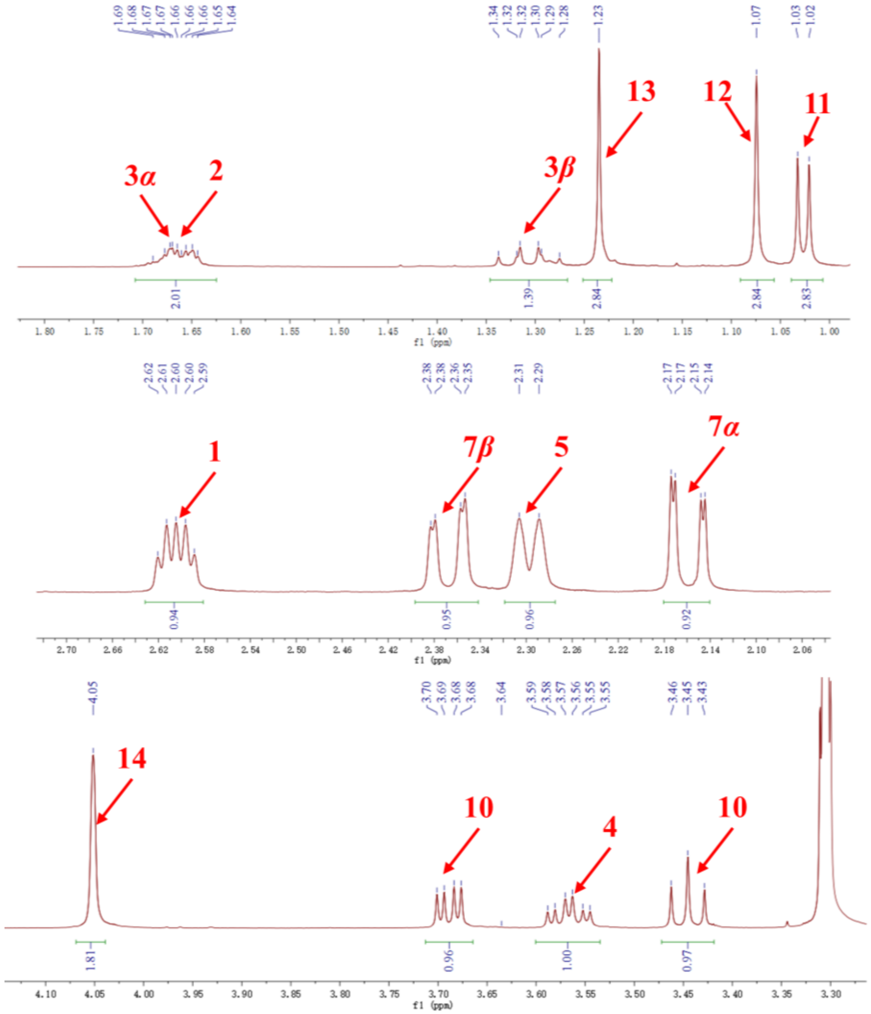

Figure S18. ^{1}H NMR spectrum of arthrinin F (**2**) (CD_3OD).

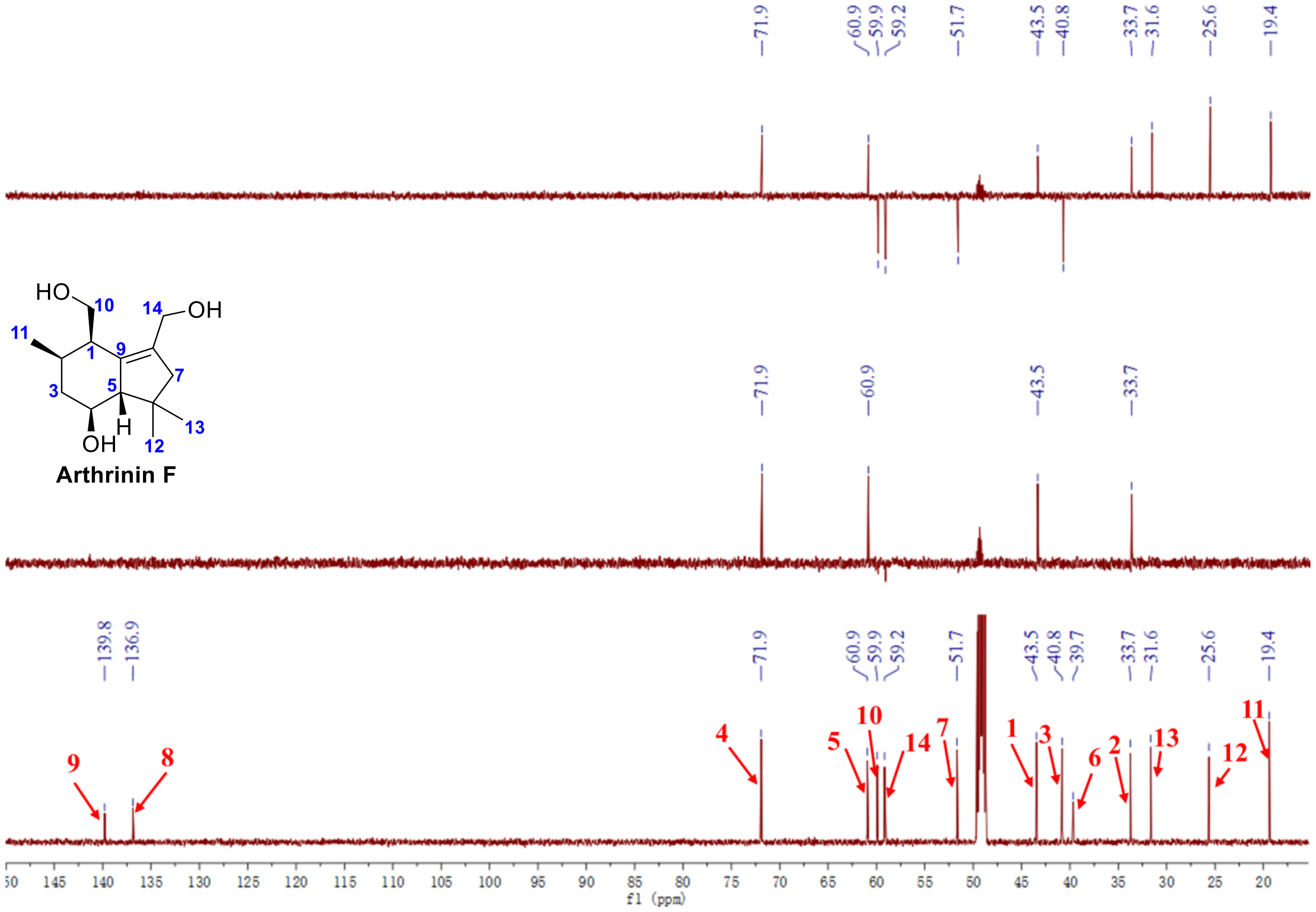

Figure S19. ^{13}C NMR and DEPT spectrum of arthrinin F (**2**) (CD_3OD).

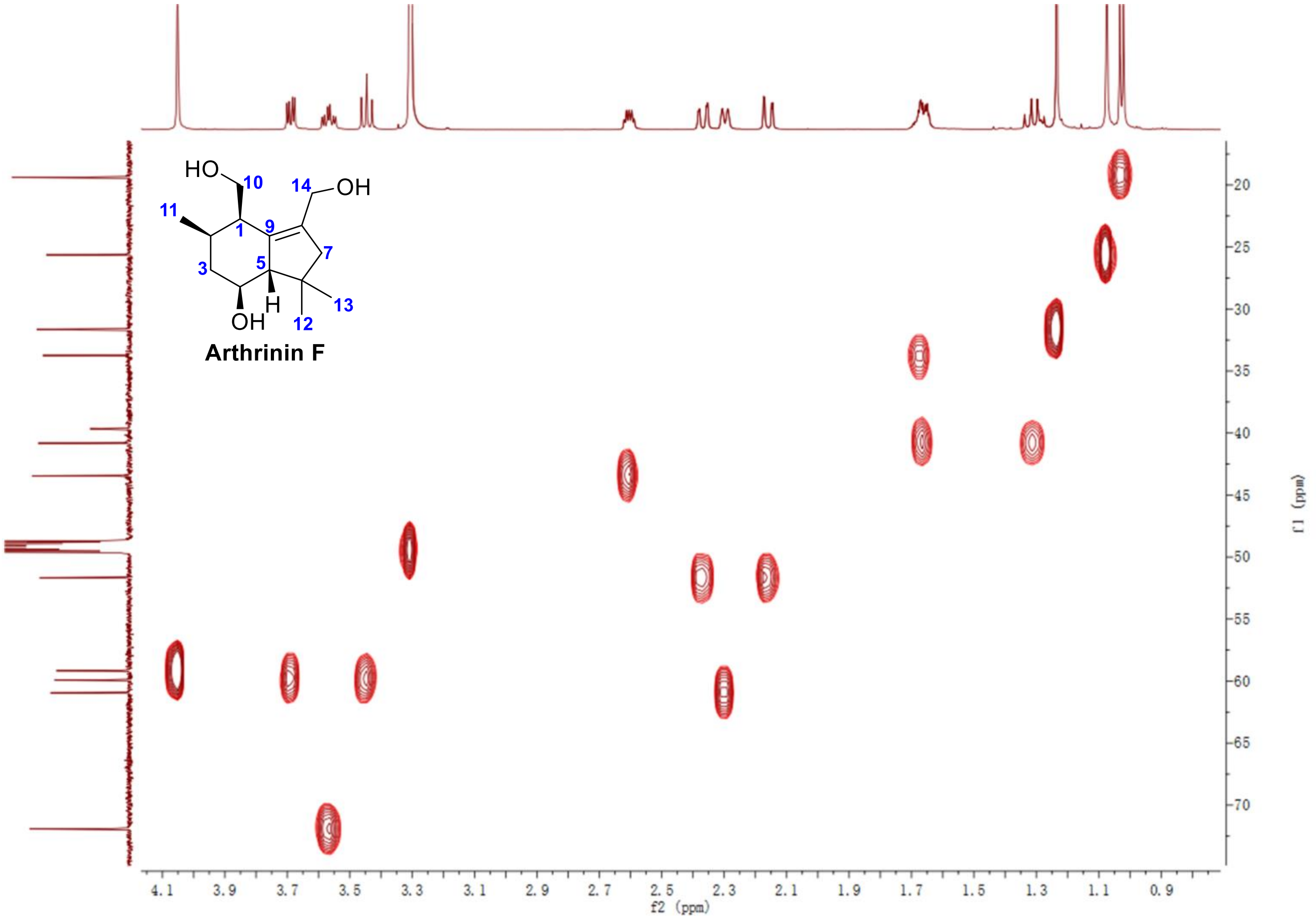

Figure S20. HSQC spectrum of arthrinin F (**2**) (CD_3OD).

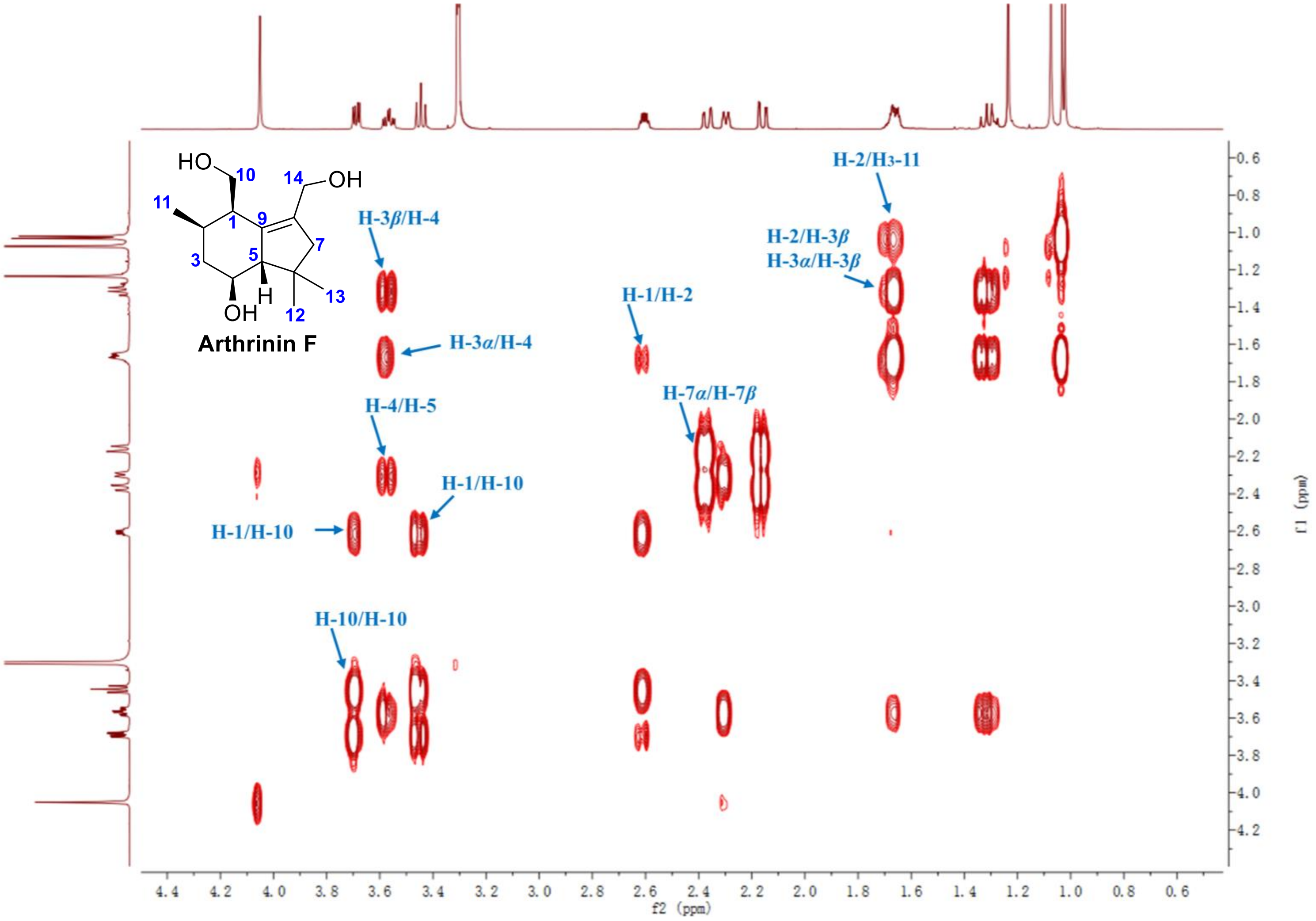

Figure S21. ^{1}H-^{1}H COSY spectrum of arthrinin F (**2**) (CD_3OD) and the selected cross-peaks (blue arrow and text).

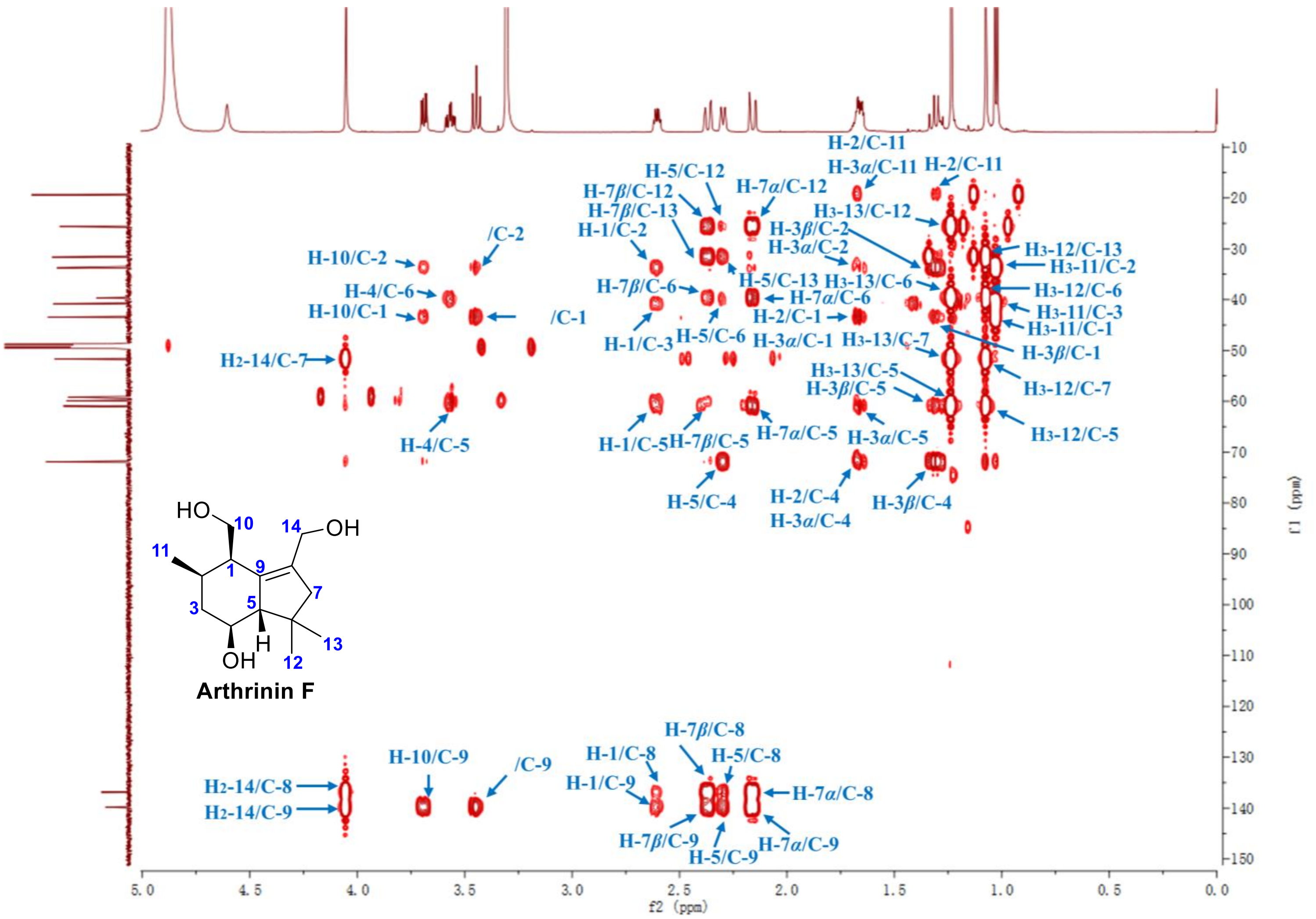

Figure S22. HMBC spectrum of arthrinin F (**2**) (CD_3OD) and the selected correlations (blue arrow and text).

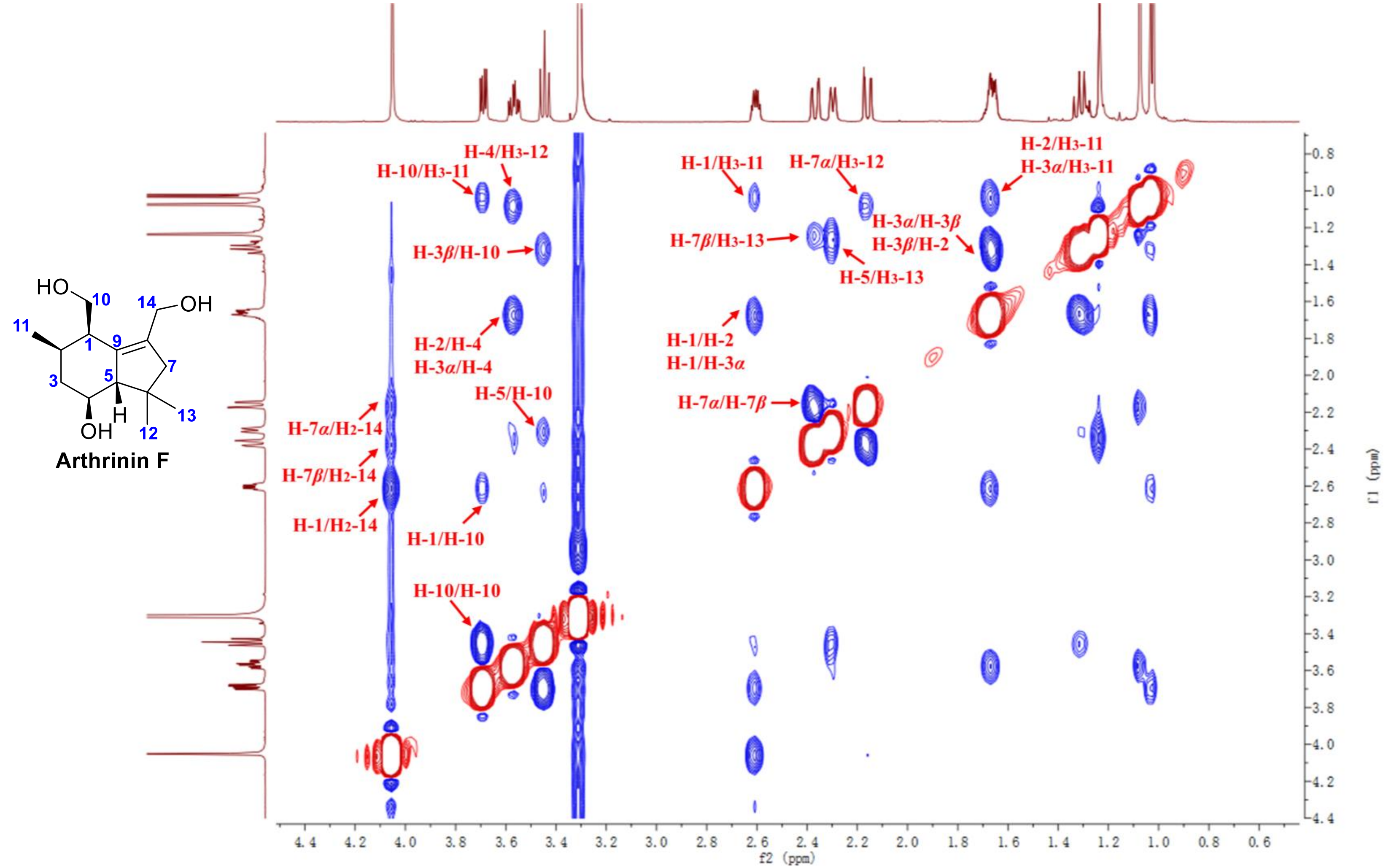

Figure S23. ROESY spectrum of arthrinin F (**2**) (CD_3OD) and the selected correlations (red arrow and text).

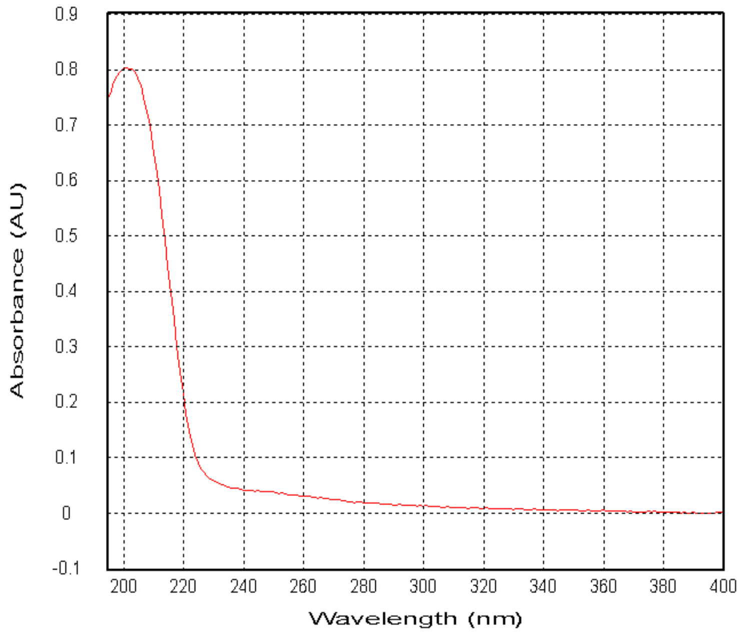

Figure S24. UV spectrum of arthrinin F (**2**).

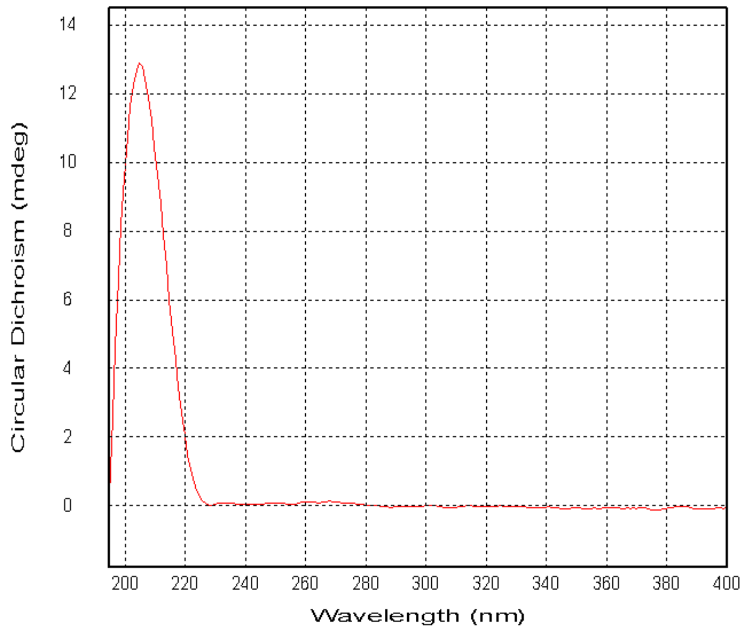

Figure S25. CD spectrum of arthrinin F (**2**).

n	Average	Std.Dev.	% RSD	Maximum	Minimum
5	107.00	0.74	0.69	108.33	106.67

S.No	Sample ID	Time	Result	Scale	OR °Arc	WLG.nm	Lg.mm	Conc.g/100ml	Temp.
1	SHSJ10b	04:35:04 PM	106.67	SR	0.064	589	100.00	0.060	22.5
2	SHSJ10b	04:35:10 PM	106.67	SR	0.064	589	100.00	0.060	22.4
3	SHSJ10b	04:35:16 PM	108.33	SR	0.065	589	100.00	0.060	22.4
4	SHSJ10b	04:35:22 PM	106.67	SR	0.064	589	100.00	0.060	22.4
5	SHSJ10b	04:35:27 PM	106.67	SR	0.064	589	100.00	0.060	22.4

Figure S26. OR report of arthrinin F (**2**).

**Sample Group**		**Info.**
**Acquisition SW Version**	6200 series TOF/6500 series Q-TOF B.05.01 (B5125.2)	

User Spectra

Fragmentor Voltage	Collision Energy	Ionization Mode
200	0	ESI

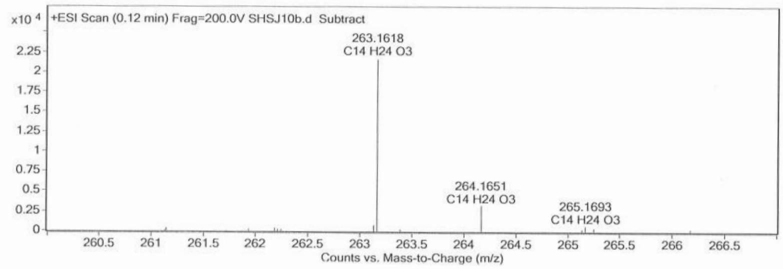

Peak List

m/z	z	Abund	Formula	Ion
119.0855	1	5423.87		
145.1008		3192.99		
175.1479		4392.33		
187.148	1	3161.62		
205.1587	1	3847.78		
263.1618	1	21597.61	C14 H24 O3	(M+Na)+
267.1573	1	3985.24		
413.2666	1	5714.33		
437.1934	1	6127.32		
709.2045	1	3395.35		

Formula Calculator Element Limits

Element	Min	Max
C	3	60
H	0	120
O	0	30

Formula Calculator Results

Formula	CalculatedMass	CalculatedMz	Mz	Diff. (mDa)	Diff. (ppm)	DBE
C14 H24 O3	240.1725	263.1618	263.1618	0.00	0.00	3.0000

Figure S27. HRESIMS spectrum of arthrinin F (**2**)

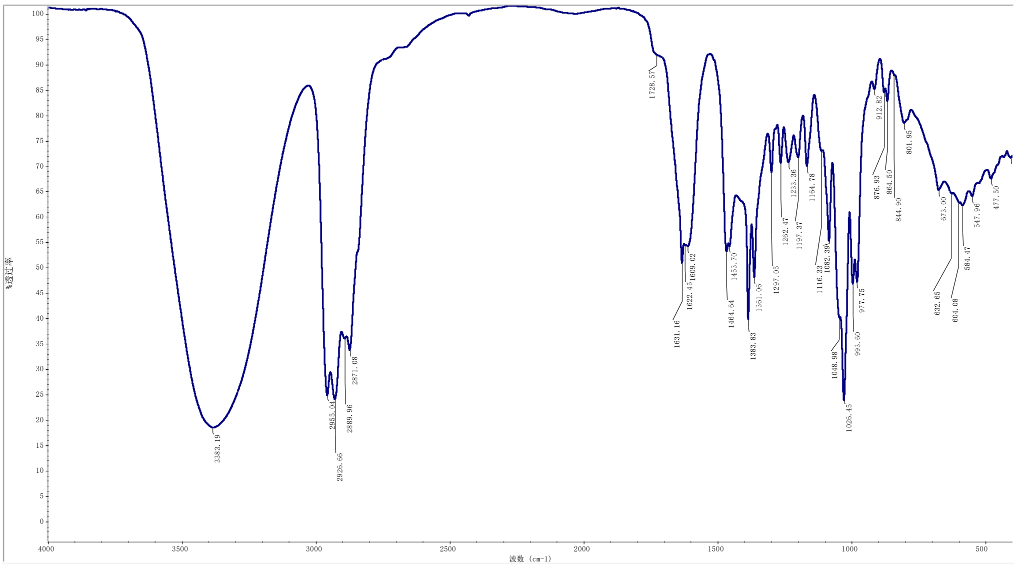

Figure S28. IR spectrum of arthrinin F (**2**).

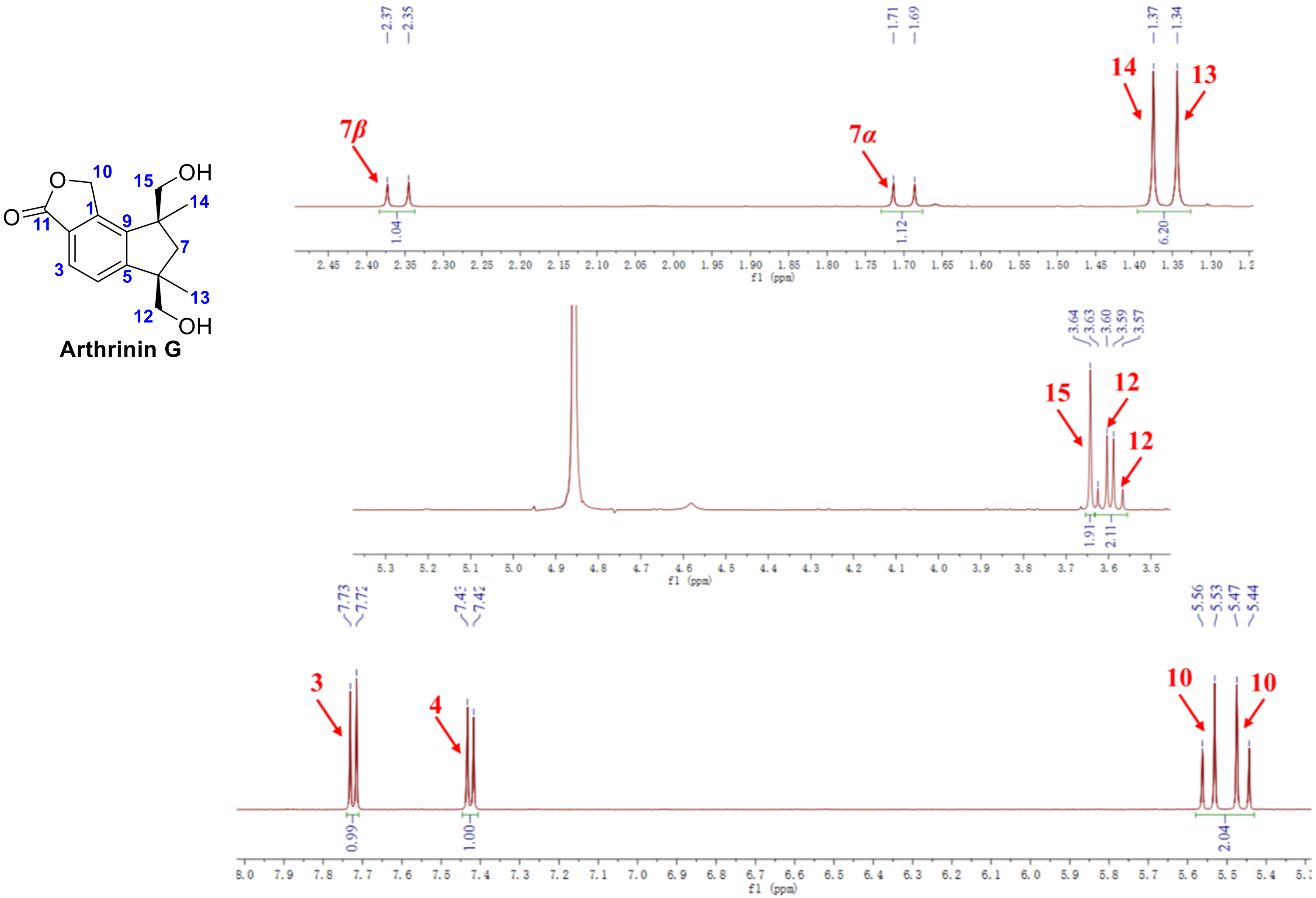

Figure S29. ^{1}H NMR spectrum of arthrinin G (**3**) (CD_3OD).

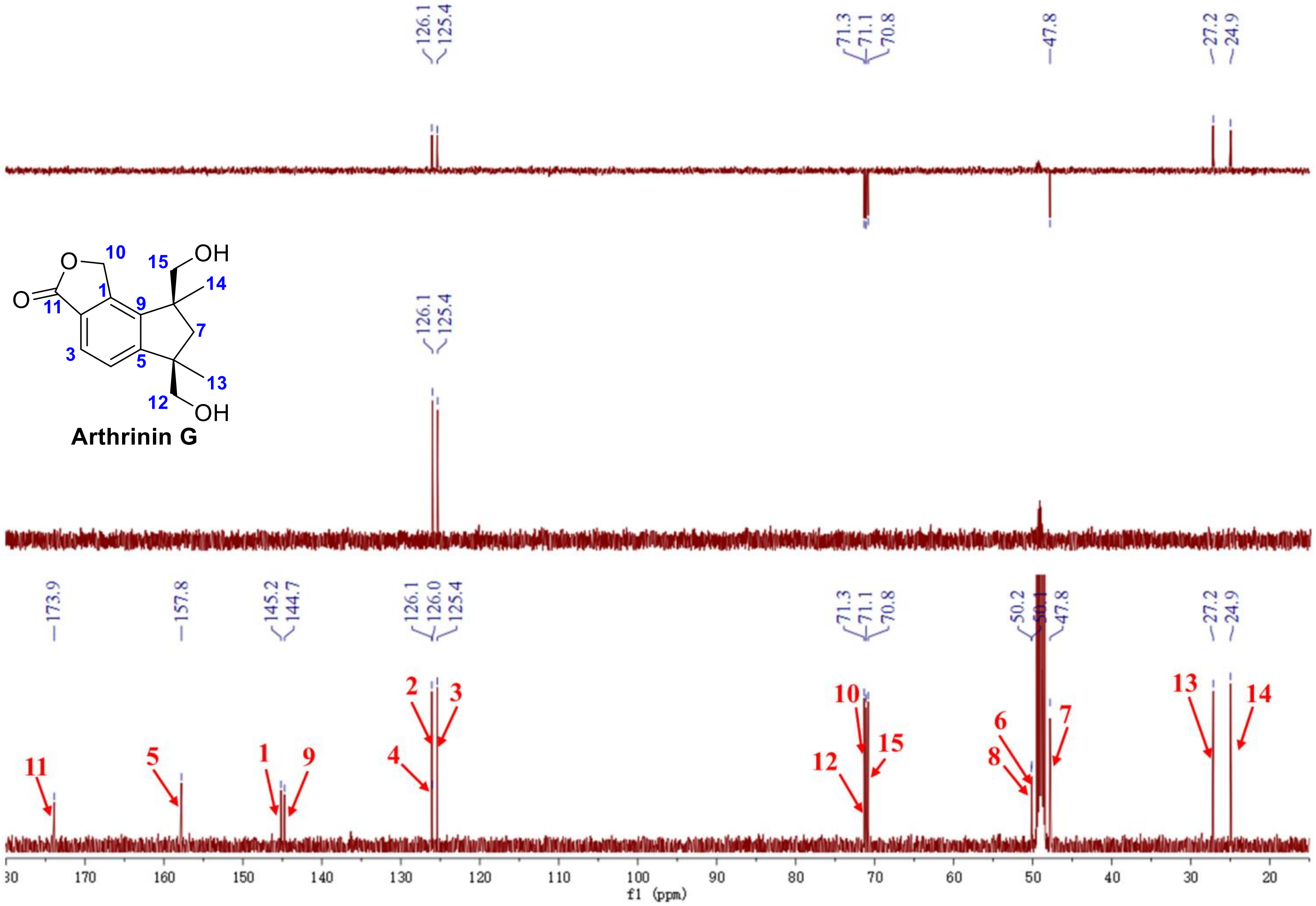

Figure S30. ^{13}C NMR and DEPT spectrum of arthrinin G (**3**) (CD$_3$OD).

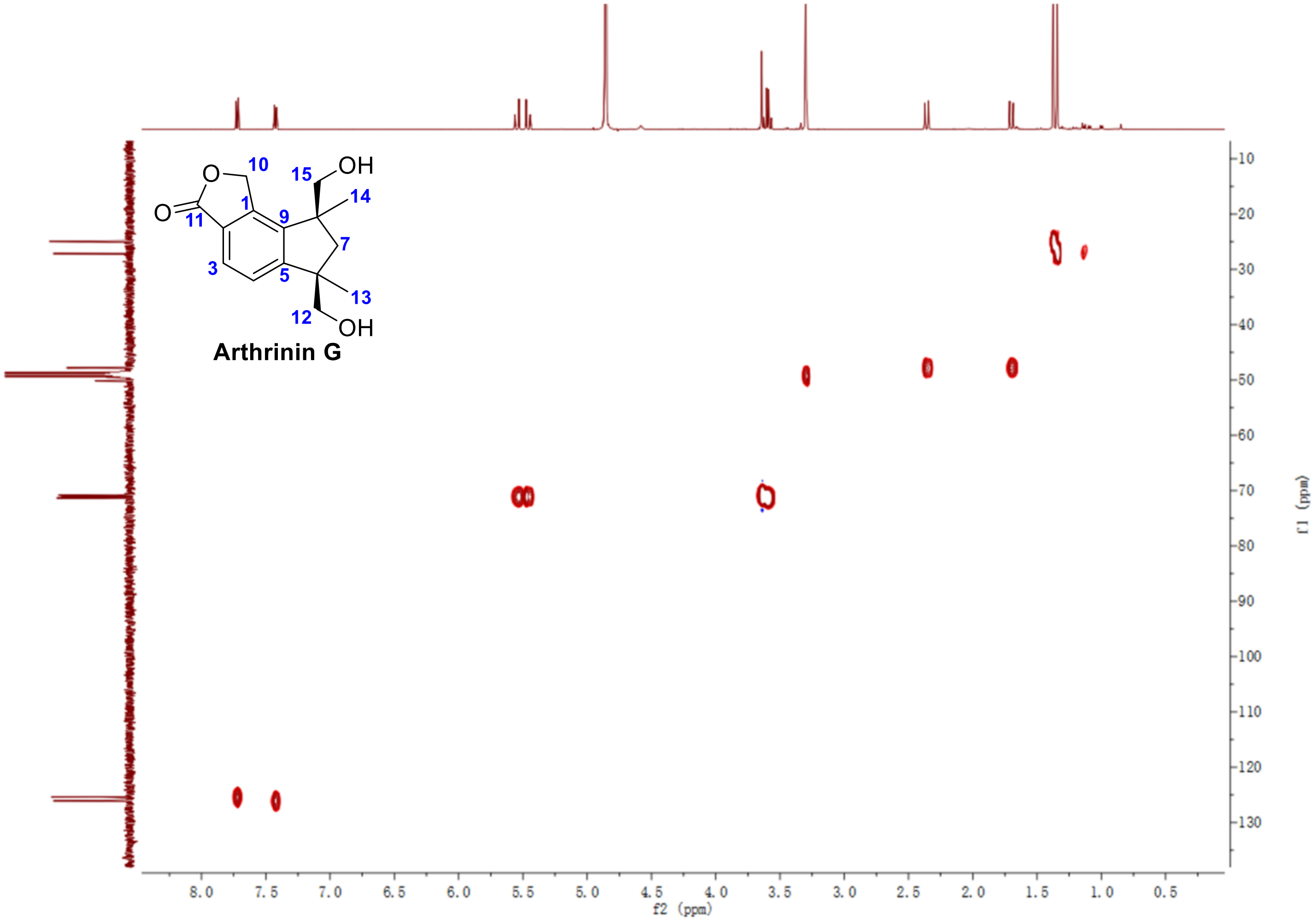

Figure S31. HSQC spectrum of arthrinin G (**3**) (CD_3OD).

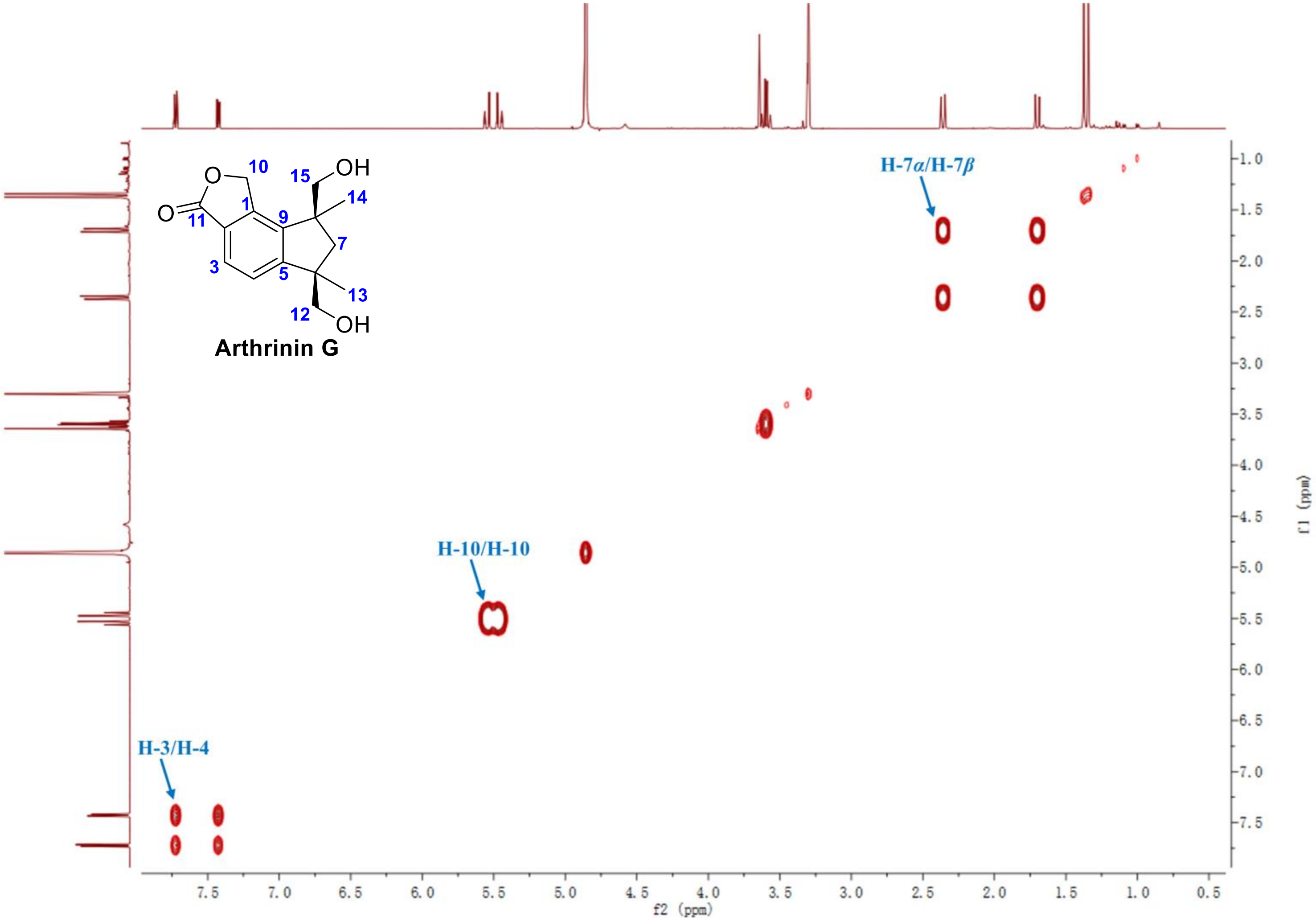

Figure S32. ${}^{1}H$-${}^{1}H$ COSY spectrum of arthrinin G (**3**) (CD_3OD) and the selected cross-peaks (blue arrow and text).

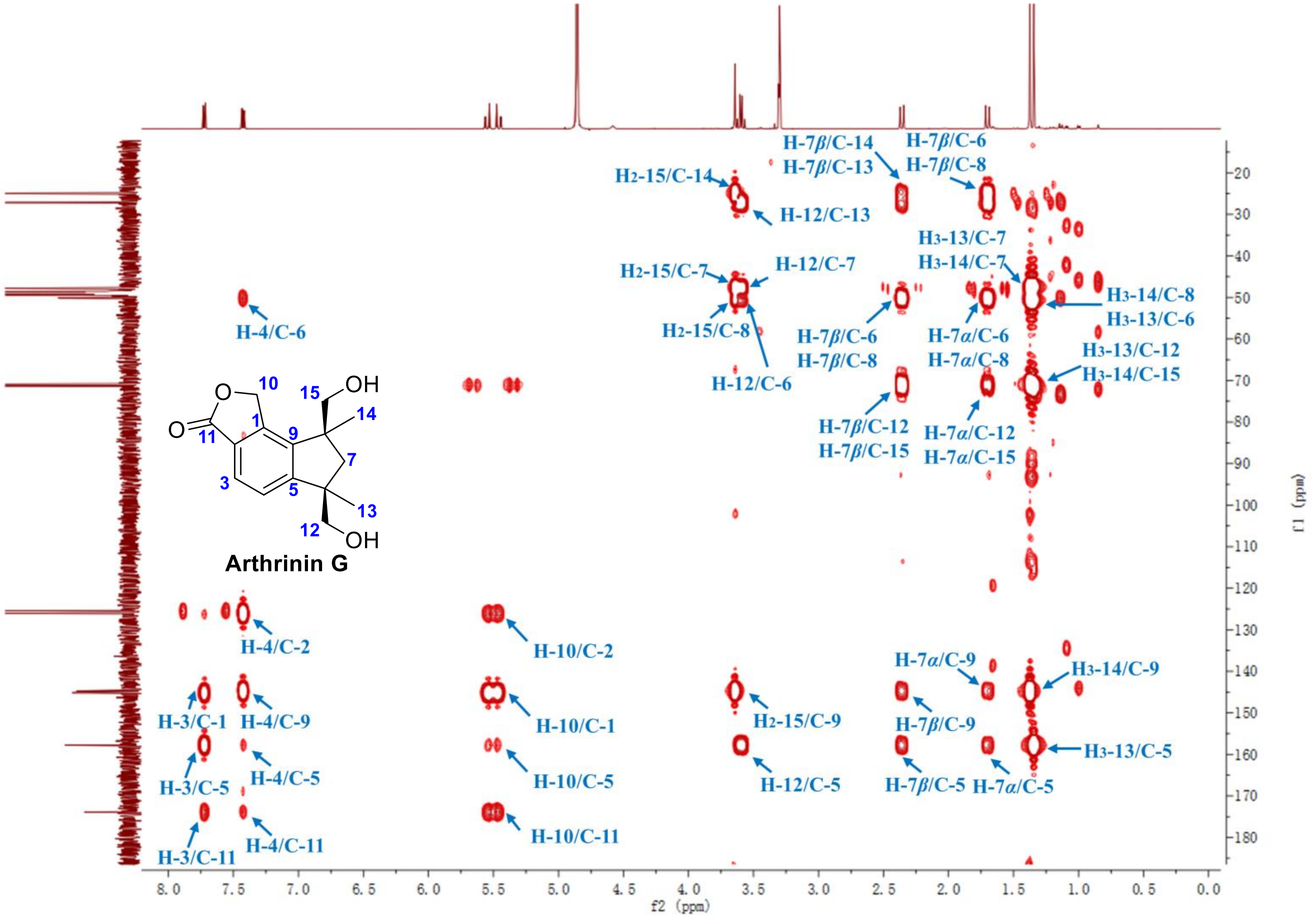

Figure S33. HMBC spectrum of arthrinin G (**3**) (CD_3OD) and the selected correlations (blue arrow and text).

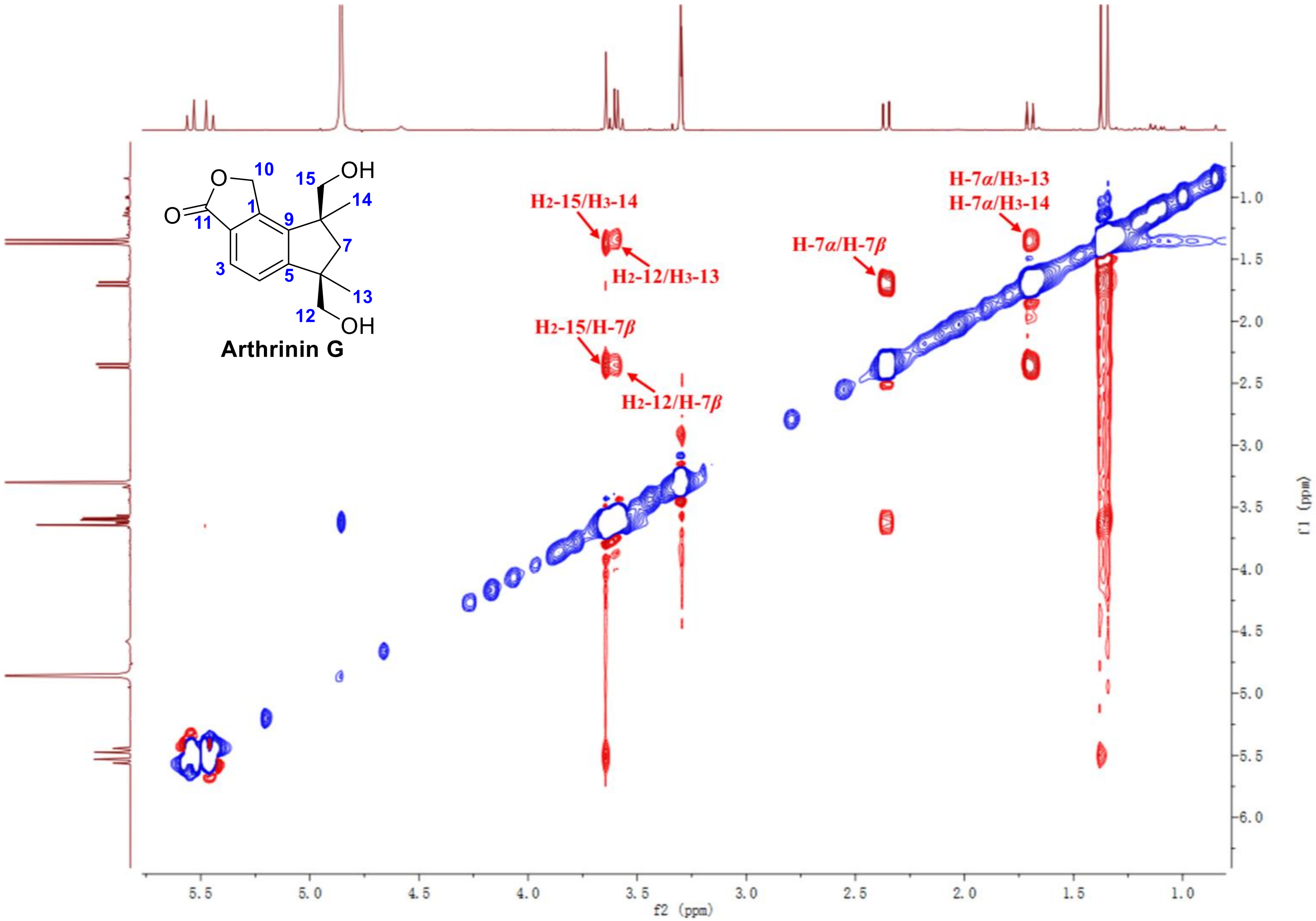

Figure S34. ROESY spectrum of arthrinin G (**3**) (CD_3OD) and the selected correlations (red arrow and text).

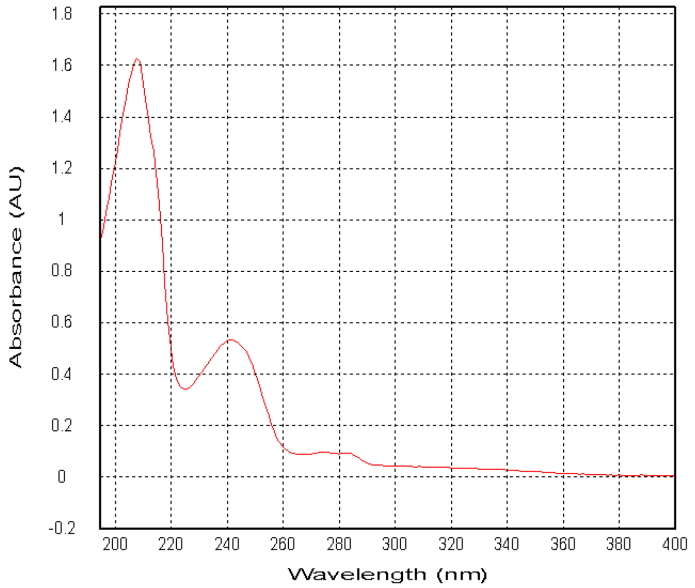

Figure S35. UV spectrum of arthrinin G (**3**).

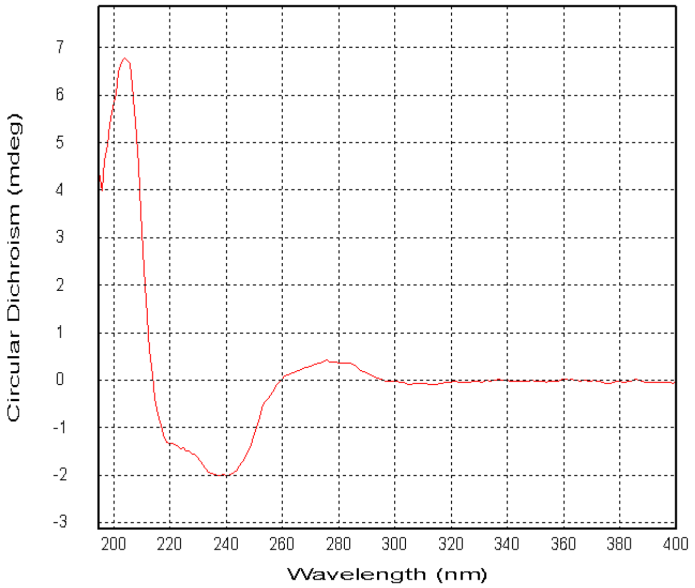

Figure S36. CD spectrum of arthrinin G (**3**).

n	Average	Std.Dev.	% RSD	Maximum	Minimum
5	12.55	0.41	3.26	12.73	11.82

S.No	Sample ID	Time	Result	Scale	OR °Arc	WLG.nm	Lg.mm	Conc.g/100ml	T
1	SHSJ05	04:18:26 PM	11.82	SR	0.013	589	100.00	0.110	2[illegible]
2	SHSJ05	04:18:32 PM	12.73	SR	0.014	589	100.00	0.110	2[illegible]
3	SHSJ05	04:18:37 PM	12.73	SR	0.014	589	100.00	0.110	2[illegible]
4	SHSJ05	04:18:43 PM	12.73	SR	0.014	589	100.00	0.110	2[illegible]
5	SHSJ05	04:18:49 PM	12.73	SR	0.014	589	100.00	0.110	2[illegible]

Figure S37. OR report of arthrinin G (**3**).

Sample Group **Info.**

Acquisition SW 6200 series TOF/6500 series

Version Q-TOF B.05.01 (B5125.2)

User Spectra

Fragmentor Voltage	Collision Energy	Ionization Mode
200	0	ESI

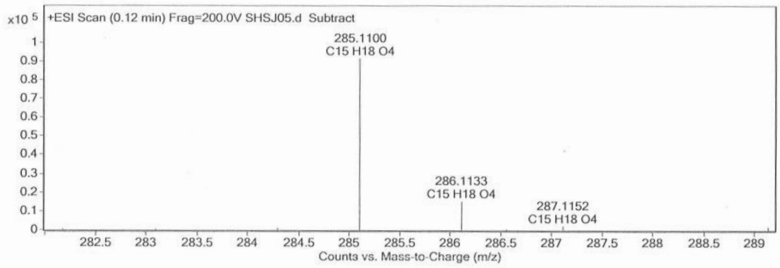

Peak List

m/z	z	Abund	Formula	Ion
263.1275	1	49214.64		
285.11	1	91415.52	C15 H18 O4	(M+Na)+
286.1133	1	15192.29	C15 H18 O4	(M+Na)+
311.183	1	14938.17		
320.1473	1	38863.32		
326.1368	1	18726.91		
333.131	1	65233.94		
405.1888	1	27077.16		
547.2306	1	65405.82		
548.2337	1	21594.29		

Formula Calculator Element Limits

Element	Min	Max
C	3	60
H	0	120
O	0	30

Formula Calculator Results

Formula	CalculatedMass	CalculatedMz	Mz	Diff. (mDa)	Diff. (ppm)	DBE
C15 H18 O4	262.1205	285.1097	285.1100	-0.30	-1.05	7.0000

Figure S38. HRESIMS spectrum of arthrinin G (**3**)

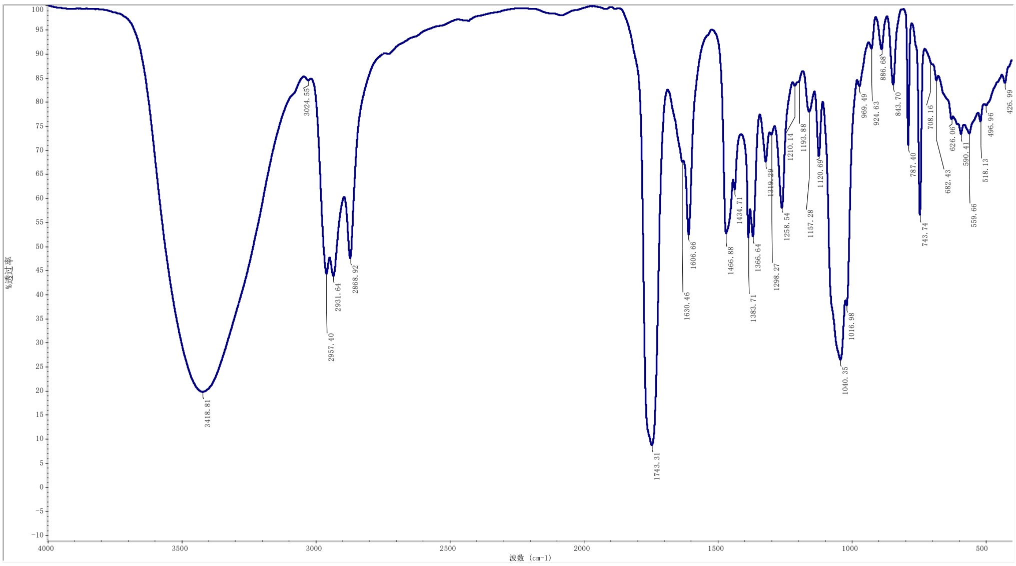

Figure S39. IR spectrum of arthrinin G (**3**).

Table S6. Conformational analysis of the B3LYP-D3BJ-SCRF/6-31G(d) optimized conformers of (1*R**, 2*R**, 4*S**, 5*S**)-**2** in MeOH with SMD solvent model (T=298.15 K)

Conformer	E (Hartree)[a]	C (Hartree)[b]	G (kcal/mol)[c]	ΔG (kcal/mol)[d]	Population[e]
**2a**	-773.934134	0.32164	-485441.839992	0.0	45.55%
**2b**	-773.934967	0.322714	-485441.688598	0.151394	35.27%
**2c**	-773.934926	0.324232	-485440.710641	1.129351	6.76%
**2d**	-773.934922	0.324409	-485440.596815	1.243177	5.58%
**2e**	-773.935178	0.324937	-485440.426218	1.413774	4.18%
**2f**	-773.934036	0.324222	-485440.158294	1.681697	2.66%

[a] Electronic energy obtained at B3LYP-D3BJ-SCRF/6-311+G(d,p) level of theory;

[b] Thermal correction to Gibbs free energy obtained at B3LYP-D3BJ-SCRF/6-31G(d) level of theory; [c] Gibbs free energy (E + C);

[d] The relative Gibbs free energy;

[e] The Boltzmann distribution of each conformer.

Table S7. Cartesian coordinates (Å) of **2a** obtained at the B3LYP-D3BJ-SCRF/6-31G(d) level of theory in MeOH with SMD solvent model.

C	-1.554525	0.699587	-0.133616	H	0.057635	-1.985604	-1.419080
C	-2.199237	-0.497909	-0.880617	H	-1.734222	1.603698	-0.728172
C	-1.750272	-1.843215	-0.286815	H	3.054752	1.152736	-0.311632
C	-0.232694	-2.008183	-0.364431	H	2.535015	1.067873	1.368585
C	0.474178	-0.881331	0.395176	H	2.612896	-2.603413	1.172801
C	2.024585	-0.740034	0.208740	H	3.882978	-1.365156	1.163083
C	2.249610	0.793742	0.340947	H	2.512873	-1.221646	2.279227
C	0.910995	1.408576	0.001868	H	1.939284	-0.626940	-1.975898
C	-0.071461	0.489716	0.028080	H	3.543974	-0.986938	-1.318629
C	-2.191308	0.964094	1.248726	H	2.305676	-2.250562	-1.356817
C	-3.725272	-0.398840	-0.965412	H	-3.251329	1.202636	1.134212
C	2.802569	-1.528179	1.266492	H	-2.111080	0.076393	1.891181
C	2.471927	-1.180604	-1.193419	H	-0.659860	1.920473	1.975313
C	0.793484	2.871433	-0.297297	H	-4.200548	-0.558925	0.009348
O	0.184810	-3.303896	0.096413	H	-4.041188	0.583786	-1.337224
O	-1.613282	2.100090	1.896652	H	-4.118667	-1.158706	-1.650908
O	1.593064	3.247834	-1.435138	H	-0.018949	-3.346565	1.047012
H	0.263890	-1.064004	1.463978	H	-0.251412	3.168918	-0.445291
H	-1.807997	-0.456125	-1.907679	H	1.190346	3.465356	0.534801
H	-2.224060	-2.667432	-0.834273	H	1.260326	2.731511	-2.188858

H	-2.072542	-1.926695	0.760976	-	-	-	-

Table S8. Cartesian coordinates (Å) of **2b** obtained at the B3LYP-D3BJ-SCRF/6-31G(d) level of theory in MeOH with SMD solvent model.

C	-1.586975	-0.320533	-0.301889	H	1.298785	-1.881102	-1.143997
C	-1.427040	-1.797583	-0.754389	H	-2.229325	0.185202	-1.033990
C	-0.346162	-2.516255	0.068860	H	2.036300	2.509940	-0.968261
C	1.014593	-1.836160	-0.083981	H	1.518981	2.550688	0.713185
C	0.944533	-0.378203	0.367023	H	3.981007	1.299882	1.010395
C	2.172792	0.546190	0.053279	H	2.675293	0.909534	2.143941
C	1.508624	1.931830	-0.197330	H	3.615871	-0.392446	1.393768
C	0.081862	1.612445	-0.574991	H	3.706630	0.817857	-1.457106
C	-0.237639	0.350181	-0.249748	H	3.347955	-0.883086	-1.120758
C	-2.296509	-0.179942	1.053971	H	2.219895	0.090324	-2.087463
C	-2.742284	-2.582177	-0.771752	H	-3.216633	-0.774780	1.065970
C	3.167912	0.591876	1.216789	H	-1.653582	-0.528039	1.871036
C	2.900343	0.110801	-1.228004	H	-2.928617	1.309653	2.172033
C	-0.866835	2.675664	-1.042615	H	-3.095673	-2.815253	0.239168
O	2.022629	-2.489290	0.705739	H	-3.533826	-2.022693	-1.285569
O	-2.636912	1.203905	1.251980	H	-2.608571	-3.534239	-1.298821
O	-1.379140	3.431844	0.069879	H	2.105917	-3.398814	0.372791
H	0.800700	-0.411761	1.460351	H	-0.356475	3.397427	-1.689787
H	-1.061665	-1.746242	-1.790248	H	-1.694038	2.238763	-1.619504
H	-0.263341	-3.559975	-0.263702	H	-1.820142	2.758375	0.632392
H	-0.621345	-2.539682	1.132051	-	-	-	-

Table S9. Key transitions, oscillator strengths, and rotatory strengths in the ECD spectrum of conformer (1*R*, 2*R*, 4*S*, 5*S*)-**2a** at the CAM-B3LYP-SCRF/def2-SVP level of theory in MeOH with SMD solvent model.

Num[a]	Transition[b]	CI-coeff[c]	ΔE (eV)[d]	λ (nm)[e]	f[f]	R_{vel}[g]	R_{len}[h]
1	66->67	0.69898	6.4549	192.08	0.4435	21.2461	22.3192
2	63->67	-0.43662	7.1246	174.02	0.0080	-3.3436	-9.5176
	64->67	0.5033					
3	66->68	0.6415	7.3969	167.62	0.0134	3.4553	6.2095
4	65->67	0.62924	7.5889	163.38	0.0035	-4.5174	-3.5602
5	66->69	0.62741	7.7636	159.70	0.0388	-55.0972	-55.4109
6	63->67	0.51702	7.7820	159.32	0.0175	5.3234	5.4209
	64->67	0.44667					
7	62->67	0.61815	7.8927	157.09	0.0097	33.6284	38.4606
8	65->68	0.26302	8.0670	153.69	0.0047	4.4293	3.0968
	65->69	-0.22886					

	66->71	-0.25786					
9	56->67	0.23836	8.2123	150.97	0.0063	35.9523	28.8258
	57->67	0.25571					
	59->67	0.33868					
	61->67	-0.22913					
10	63->68	0.26746	8.2716	149.89	0.0032	-7.3463	-7.7196
	64->68	0.33682					
11	58->67	0.27857	8.3674	148.18	0.0008	-15.7591	-9.3366
	59->67	0.24784					
	64->69	0.23495					
12	66->70	0.56412	8.4282	147.11	0.0057	-11.8385	-28.5102
13	66->71	0.5287	8.5098	145.70	0.0124	8.3824	8.7162
	66->73	-0.24402					
14	60->67	-0.29988	8.6665	143.06	0.0049	-4.2685	-1.8769
	61->67	0.43866					
15	66->72	0.52452	8.7696	141.38	0.0179	5.4326	5.2356
16	66->73	0.56136	8.8422	140.22	0.0151	26.4489	24.9091
17	58->67	0.23758	8.8827	139.58	0.0641	-17.39	-17.4392
	60->67	0.53812					
18	58->67	0.46965	8.9392	138.70	0.0020	-4.089	-2.4803
	61->67	-0.2797					
19	65->68	0.27407	9.0000	137.76	0.0055	17.7371	17.2238
	65->69	0.40972					
	66->72	0.3066					
20	55->67	0.38986	9.1045	136.18	0.0081	13.1156	15.48
	56->67	-0.28149					
21	55->67	-0.23746	9.1268	135.85	0.0081	20.1441	24.0625
	64->68	0.23903					
	65->68	0.26387					
	66->74	-0.26084					
22	63->68	0.28353	9.1924	134.88	0.0183	25.2529	27.2204
	64->69	0.30057					
	66->75	-0.2808					
23	63->69	0.26602	9.2053	134.69	0.0020	-3.3024	2.5784
	65->71	0.23884					
24	66->75	0.46677	9.2356	134.25	0.0402	-44.798	-44.0284
25	56->67	-0.26661	9.2670	133.79	0.0115	-17.4674	-17.4577
	57->67	-0.34192					
	59->67	0.40631					
26	65->70	-0.25099	9.2879	133.49	0.0249	6.1903	4.732

	66->74	0.34743					
27	63->69	0.26549	9.3125	133.14	0.0068	8.7549	13.2764
	64->69	0.24523					
	65->70	0.30146					
28	55->67	0.28566	9.4117	131.73	0.0094	8.9253	10.7588
	56->67	0.26288					
	57->67	-0.27813					
	62->68	0.24067					
	64->70	-0.26525					
29	55->67	0.26625	9.4272	131.52	0.0166	-25.9594	-27.461
	56->67	0.25827					
	64->70	0.29192					
30	62->68	0.41981	9.5447	129.90	0.0420	48.552	42.9175
	62->69	-0.27121					

[a] Number of the excited states; [b] Only transitions with contribution over 10.0% were listed; [c] Configuration-interaction coefficient; [d] Excitation energy; [e] Wavelength; [f] Oscillator strength; [g] Rotatory strength in length form (10^{-40} cgs); [h] Rotatory strength in velocity form (10^{-40} cgs).

Table S10. Key transitions, oscillator strengths, and rotatory strengths in the ECD spectrum of conformer (1*R*, 2*R*, 4*S*, 5*S*)-**2b** at the CAM-B3LYP-SCRF/def2-SVP level of theory in MeOH with SMD solvent model.

Num[a]	Transition[b]	CI-coeff[c]	ΔE (eV)[d]	λ (nm)[e]	f[f]	R_{vel}[g]	R_{len}[h]
1	66->67	0.69748	6.5024	190.67	0.4158	28.7686	32.7882
2	64->67	-0.29407	7.0828	175.05	0.0119	-25.717	-21.8508
	65->67	0.6145					
3	66->68	0.52826	7.7632	159.71	0.0247	-0.7411	0.3094
	66->69	0.39504					
4	62->67	0.24372	7.8128	158.69	0.0018	-8.5608	-7.4167
	63->67	0.41739					
	64->67	0.40332					
5	66->68	-0.34027	7.8646	157.65	0.0084	-17.707	-18.3355
	66->69	0.53972					
	66->71	-0.22885					
6	61->67	0.46685	7.9549	155.86	0.0078	30.9383	29.9705
	63->67	-0.32022					
	64->67	0.27505					
7	64->68	0.36128	8.0515	153.99	0.0096	6.2912	27.8722
	64->71	-0.28529					
	65->68	0.21789					
8	61->67	-0.3061	8.1050	152.97	0.0034	8.5324	-2.7092

	63->69	0.36094					
9	61->67	0.22305	8.1517	152.10	0.0072	-1.9317	16.98
	63->67	0.26109					
	63->69	0.40515					
10	66->70	0.58456	8.3653	148.21	0.0081	3.1405	4.4764
	66->72	-0.2653					
11	62->67	0.24236	8.4848	146.13	0.0122	-2.2965	-5.2042
	66->71	0.44336					
	66->72	-0.23445					
12	62->67	0.49652	8.5222	145.48	0.0014	-4.3322	-4.9824
	64->67	-0.21705					
	66->71	-0.23557					
13	56->67	-0.23935	8.5698	144.68	0.0073	-1.0272	-1.7964
	59->67	-0.27924					
	66->72	0.36811					
14	56->67	0.27099	8.6027	144.12	0.0288	29.6694	32.5035
	59->67	0.31967					
	63->67	0.21919					
	66->72	0.34844					
15	56->67	0.30678	8.7227	142.14	0.0062	-16.9215	-19.2292
	58->67	0.3658					
	59->67	-0.25812					
16	60->67	-0.32238	8.8779	139.65	0.0039	-17.5155	-12.786
	65->68	0.22817					
	66->73	0.33919					
17	60->67	0.31205	8.8840	139.56	0.0130	-4.6921	-4.6448
	66->73	0.46681					
18	58->67	-0.21601	8.9214	138.97	0.0100	6.3945	7.4684
	60->67	0.46986					
19	66->71	0.2319	9.0246	137.38	0.0043	1.3981	2.6957
	66->73	0.27171					
	66->74	0.50814					
20	55->67	0.21303	9.0994	136.25	0.0164	4.8322	7.8352
	56->67	-0.23026					
	58->67	0.36129					
	59->67	0.3777					
21	65->68	-0.21875	9.1422	135.62	0.0097	0.995	-12.5654
	65->69	0.42754					
22	64->68	0.32751	9.2443	134.12	0.0664	15.3327	17.8446
	64->70	0.26164					

	65->69	0.24426					
23	66->75	0.43187	9.2607	133.88	0.0342	-56.7389	-57.701
	66->76	-0.29981					
24	64->69	0.45153	9.2837	133.55	0.0403	-48.9783	-49.2497
	66->75	0.24804					
25	55->67	-0.26277	9.3241	132.97	0.0041	16.2571	12.0476
	57->67	0.49717					
26	63->68	0.39456	9.3844	132.12	0.0101	-4.833	-1.5909
	63->70	0.3202					
27	62->68	0.30335	9.4356	131.40	0.0659	35.2476	28.662
	63->70	-0.24962					
	65->70	-0.29891					
28	66->75	0.30655	9.5060	130.43	0.0042	12.5287	11.8301
	66->76	0.44874					
29	62->68	0.21465	9.5789	129.43	0.0041	5.4779	3.0115
30	55->67	0.51979	9.6146	128.95	0.0153	-10.3941	-8.965
	56->67	0.23554					
	57->67	0.26269					

[a] Number of the excited states; [b] Only transitions with contribution over 10.0% were listed; [c] Configuration-interaction coefficient; [d] Excitation energy; [e] Wavelength; [f] Oscillator strength; [g] Rotatory strength in length form (10^{-40} cgs); [h] Rotatory strength in velocity form (10^{-40} cgs).

Table S11. Conformational analysis of the B3LYP-D3BJ-SCRF/6-31G(d) optimized conformers of (6*R**, 8*S**)-**3** in MeOH with SMD solvent model (T=298.15 K)

Conformer	E (Hartree)[a]	C (Hartree)[b]	G (kcal/mol)[c]	ΔG (kcal/mol)[d]	Population[e]
**3a**	-883.707001	0.264403	-554360.230552	0.0	31.30%
**3b**	-883.708058	0.265711	-554360.072931	0.157622	23.98%
**3c**	-883.705846	0.263837	-554359.86093	0.369623	16.77%
**3d**	-883.708595	0.267049	-554359.56994	0.660613	10.26%
**3e**	-883.705364	0.264228	-554359.312913	0.917639	6.64%
**3f**	-883.705294	0.264673	-554358.989848	1.240705	3.85%
**3g**	-883.705723	0.26514	-554358.965883	1.26467	3.70%
**3h**	-883.706072	0.265539	-554358.934423	1.296129	3.51%

[a] Electronic energy obtained at B3LYP-D3BJ-SCRF/6-311+G(d,p) level of theory;
[b] Thermal correction to Gibbs free energy obtained at B3LYP-D3BJ-SCRF/6-31G(d) level of theory; [c] Gibbs free energy (E + C);
[d] The relative Gibbs free energy;
[e] The Boltzmann distribution of each conformer.

Table S12. Cartesian coordinates (Å) of **3a** obtained at the B3LYP-D3BJ-SCRF/6-31G(d) level of theory in MeOH with SMD solvent model.

C	-1.359194	0.318129	-0.150472	H	1.642075	-1.402853	2.121074
C	-2.051215	-0.890016	-0.041434	H	4.018410	-1.337002	0.589576
C	-1.408609	-2.128561	-0.040817	H	2.807448	-2.629162	0.513039
C	-0.019253	-2.140281	-0.153384	H	2.433186	-2.326954	-1.913200
C	0.682069	-0.930128	-0.259092	H	2.135694	-0.715038	-2.588177
C	2.186767	-0.761023	-0.405680	H	3.726327	-1.108949	-1.907742
C	2.382490	0.772113	-0.232737	H	0.098048	1.448206	2.061658
C	1.003363	1.464823	-0.429502	H	-0.155890	3.103895	0.413280
C	0.028323	0.309945	-0.261433	H	1.597126	3.300917	0.539074
C	-2.365426	1.432371	-0.105221	H	1.045947	1.341352	-2.609384
C	-3.484571	-0.594066	0.061956	H	-0.153552	2.485321	-1.980932
C	2.947783	-1.550411	0.674393	H	1.571147	2.907388	-1.960706
C	2.646664	-1.259008	-1.787346	H	3.132127	1.165144	-0.926306
C	0.856150	2.086100	-1.830034	H	2.728001	0.968198	0.785813
C	0.784290	2.571350	0.616957	H	-2.249564	2.088577	0.762206
O	-3.647733	0.761319	0.017911	H	-2.389750	2.041403	-1.013368
O	0.812243	2.100933	1.964323	H	-1.979840	-3.047816	0.045758
O	2.585012	-1.193153	2.008410	H	0.518009	-3.084273	-0.157511
O	-4.437364	-1.344896	0.170472	-	-	-	-

Table S13. Cartesian coordinates (Å) of **3b** obtained at the B3LYP-D3BJ-SCRF/6-31G(d) level of theory in MeOH with SMD solvent model.

C	-1.367035	0.335333	-0.192935	H	2.978284	-0.811923	2.695709
C	-2.085415	-0.850136	-0.031370	H	3.932073	-1.206410	0.713393
C	-1.473135	-2.105213	-0.009628	H	2.677171	-2.449833	0.920867
C	-0.088684	-2.152636	-0.148686	H	3.744395	-1.517295	-1.768068
C	0.638282	-0.963289	-0.308881	H	2.462820	-2.722628	-1.532219
C	2.147344	-0.847129	-0.445590	H	2.175091	-1.313502	-2.570980
C	2.380974	0.683844	-0.629162	H	1.585895	0.988305	1.941968
C	1.008447	1.426423	-0.519401	H	0.077351	3.008936	0.647535
C	0.016529	0.290137	-0.343580	H	1.845341	3.061039	0.610948
C	-2.340694	1.476399	-0.133489	H	0.758212	1.597280	-2.677453
C	-3.505027	-0.514152	0.118723	H	-0.255589	2.725364	-1.756151
C	2.851480	-1.370581	0.812221	H	1.482901	3.026650	-1.917067
C	2.662574	-1.651561	-1.650461	H	2.828622	0.876655	-1.608578
C	0.726794	2.241811	-1.792445	H	3.086649	1.059377	0.118311
C	0.977858	2.385825	0.689355	H	-2.167472	2.146336	0.713817
O	-3.634702	0.844210	0.056064	H	-2.389644	2.066817	-1.052510

O	0.947587	1.734653	1.953412	H	-2.064089	-3.006872	0.119561
O	2.349295	-0.683851	1.967075	H	0.428716	-3.107449	-0.126536
O	-4.473239	-1.236424	0.278377	-	-	-	-

Table S14. Cartesian coordinates (Å) of **3c** obtained at the @@@ level of theory in the gas phase.

C	-1.352560	0.306188	-0.163663	H	1.589798	-1.324102	2.133652
C	-2.043832	-0.901891	-0.057387	H	4.006631	-1.332512	0.665729
C	-1.399302	-2.140132	-0.057120	H	2.790350	-2.619294	0.580677
C	-0.009372	-2.148748	-0.160868	H	3.781732	-1.113728	-1.830907
C	0.690520	-0.936788	-0.263565	H	2.499411	-2.342471	-1.857319
C	2.198107	-0.762003	-0.376190	H	2.207864	-0.742163	-2.560664
C	2.381825	0.773537	-0.206767	H	0.640108	2.778052	2.496540
C	1.003449	1.456058	-0.447263	H	-0.160760	3.134692	0.289502
C	0.033224	0.300026	-0.274914	H	1.591187	3.298193	0.500806
C	-2.353940	1.421485	-0.094330	H	-0.121900	2.413620	-2.061270
C	-3.476053	-0.604849	0.055457	H	1.594967	2.864718	-2.002596
C	2.933212	-1.539122	0.731094	H	1.114103	1.272679	-2.620118
C	2.700364	-1.271230	-1.738727	H	3.147808	1.167169	-0.882054
C	0.888979	2.036097	-1.869188	H	2.699195	0.976524	0.819536
C	0.754961	2.586816	0.555110	H	-2.224063	2.055840	0.787509
O	-3.637721	0.751497	0.026189	H	-2.385187	2.049805	-0.988899
O	0.643557	2.036420	1.869751	H	-1.968760	-3.060600	0.028295
O	2.545511	-1.162611	2.052155	H	0.530255	-3.091458	-0.156567
O	-4.430127	-1.355010	0.160413	-	-	-	-

Table S15. Cartesian coordinates (Å) of **3d** obtained at the @@@ level of theory in the gas phase.

C	-1.370460	0.349545	-0.184427	H	2.239515	0.073353	1.945242
C	-2.090595	-0.835496	-0.022170	H	3.936641	-1.180507	0.677639
C	-1.480763	-2.091433	-0.000783	H	2.745561	-2.484122	0.825379
C	-0.097500	-2.143161	-0.148471	H	3.717364	-1.522797	-1.806306
C	0.632794	-0.956331	-0.309365	H	2.410847	-2.705011	-1.594343
C	2.138280	-0.848007	-0.466410	H	2.153450	-1.268503	-2.604352
C	2.375894	0.685146	-0.646438	H	0.444608	1.164054	2.175775
C	1.014140	1.433982	-0.499305	H	0.075965	2.919816	0.792754
C	0.014614	0.300510	-0.331210	H	1.833950	3.081531	0.634729
C	-2.349528	1.489141	-0.156223	H	-0.249371	2.813584	-1.647175
C	-3.511127	-0.499967	0.118619	H	1.490759	3.047081	-1.880506
C	2.860861	-1.395124	0.781896	H	0.673224	1.666001	-2.637516
C	2.632646	-1.636057	-1.690514	H	2.798425	0.879537	-1.636604
C	0.709200	2.290966	-1.738914	H	3.104505	1.058162	0.080876

C	1.017377	2.359658	0.731949	H	-2.189011	2.186228	0.671249
O	-3.642163	0.857423	0.040367	H	-2.393170	2.051920	-1.092814
O	1.247549	1.673487	1.969400	H	-2.073884	-2.991513	0.129611
O	2.359434	-0.897771	2.016614	H	0.416798	-3.099684	-0.133838
O	-4.478715	-1.221809	0.282467	-	-	-	-

Table S16. Key transitions, oscillator strengths, and rotatory strengths in the ECD spectrum of conformer (6*R*, 8*S*)-**3a** at the CAM-B3LYP-SCRF/def2-SVP level of theory in MeOH with SMD solvent model.

Num[a]	Transition[b]	CI-coeff[c]	ΔE (eV)[d]	λ (nm)[e]	f[f]	R_{vel}[g]	R_{len}[h]
1	69->71	0.44924	5.0511	245.46	0.0293	-0.2471	-0.2749
	70->71	-0.38867					
	70->72	0.2984					
2	66->71	-0.38913	5.4038	229.44	0.1698	89.9904	98.2998
	69->71	0.32332					
	70->71	0.41611					
3	66->71	0.50856	5.4222	228.66	0.1058	-90.6629	-99.1723
	69->71	0.2421					
	70->71	0.32799					
4	69->71	-0.34877	6.2973	196.89	0.5917	-24.9166	-25.272
	70->72	0.58209					
5	68->71	0.66752	6.4530	192.13	0.0578	33.2378	35.0552
6	69->72	0.64981	6.4720	191.57	0.5045	-2.8649	-1.7009
	70->71	0.22496					
7	67->71	0.54184	6.8332	181.44	0.0123	-3.3437	-1.8281
8	64->71	0.26626	7.0456	175.97	0.0433	-13.5175	-14.3769
	65->71	0.58822					
9	68->72	0.67556	7.0697	175.37	0.0231	-8.1471	-8.7024
10	62->71	-0.33774	7.1018	174.58	0.0091	4.473	4.1523
	65->71	-0.28162					
	67->71	0.36742					
11	60->71	0.29987	7.2363	171.34	0.0045	-4.8565	-5.6086
	63->71	0.44451					
	64->71	-0.34463					
12	66->72	0.60329	7.3092	169.63	0.0058	-12.9346	-13.5471
13	67->72	0.62172	7.4794	165.77	0.0065	-3.2265	-4.2988
14	62->71	0.36415	7.6141	162.84	0.0063	4.6623	4.8702
	64->71	0.39496					
15	62->72	-0.38137	7.7964	159.03	0.0024	-2.5598	-1.27
	64->72	0.40453					
	67->72	0.24256					

16	60->71	-0.2603	7.9479	156.00	0.0058	2.4577	1.6072
	61->71	0.49434					
	62->71	-0.24483					
	63->71	0.2533					
17	55->71	-0.26521	8.0086	154.81	0.0017	-0.8297	-2.2328
	61->71	0.22841					
	63->72	0.34162					
18	55->71	0.28969	8.0539	153.94	0.0101	-1.59	-2.2
	63->72	0.27494					
	65->72	0.23516					
19	65->72	-0.32993	8.1095	152.89	0.0235	14.4789	18.4607
	68->73	-0.23939					
	70->73	0.39321					
20	63->72	-0.24222	8.1343	152.42	0.0049	-1.2389	-2.0483
	65->72	0.47097					
21	55->71	0.36848	8.1638	151.87	0.0213	23.439	23.7855
	65->72	-0.22999					
22	68->73	0.30727	8.2249	150.74	0.0539	-11.1963	-10.9175
	70->73	0.2498					
	70->74	-0.23239					
23	59->71	0.32393	8.2641	150.03	0.0121	-6.592	-8.0125
	60->71	-0.27085					
	61->71	-0.23951					
	63->71	0.268					
24	68->76	-0.22953	8.3279	148.88	0.0024	-7.6291	-10.1568
	70->74	0.31096					
25	69->73	0.27723	8.3360	148.73	0.0124	-5.822	-5.5248
26	64->72	-0.25394	8.3928	147.73	0.0402	-7.8407	-7.463
	69->73	0.34757					
27	69->74	-0.25567	8.5173	145.57	0.0220	41.2282	42.1796
	70->73	0.23519					
	70->74	0.40009					
28	60->72	-0.25986	8.6058	144.07	0.0014	-1.6198	-0.635
	61->72	0.41958					
	62->72	-0.28689					
29	57->71	0.38179	8.6281	143.70	0.0097	2.0056	2.9663
	58->71	-0.23044					
	59->71	0.33221					
30	58->71	-0.23136	8.6830	142.79	0.0120	-13.3353	-13.1485
	69->73	0.29713					

	69->74	0.29198					

[a] Number of the excited states; [b] Only transitions with contribution over 10.0% were listed; [c] Configuration-interaction coefficient; [d] Excitation energy; [e] Wavelength; [f] Oscillator strength; [g] Rotatory strength in length form (10^{-40} cgs); [h] Rotatory strength in velocity form (10^{-40} cgs).

Table S17. Key transitions, oscillator strengths, and rotatory strengths in the ECD spectrum of conformer (6*R*, 8*S*)-**3b** at the CAM-B3LYP-SCRF/def2-SVP level of theory in MeOH with SMD solvent model.

Num[a]	Transition[b]	CI-coeff[c]	ΔE (eV)[d]	λ (nm)[e]	f[f]	R_{vel}[g]	R_{len}[h]
1	69->71	-0.41855	5.0467	245.67	0.0296	0.2646	0.3553
	70->71	0.43098					
	70->72	-0.27637					
2	69->71	0.43274	5.4226	228.64	0.2497	-60.9794	-67.127
	70->71	0.4644					
3	66->71	0.61484	5.4365	228.06	0.0243	53.6465	58.5125
4	69->71	-0.32452	6.3045	196.66	0.6436	17.7599	18.9768
	70->72	0.60497					
5	68->71	-0.2938	6.4609	191.90	0.3959	-24.9539	-26.2527
	69->72	0.5735					
	70->71	0.23055					
6	68->71	0.6035	6.5137	190.34	0.1292	25.0142	27.0251
	69->72	0.30267					
7	67->71	0.67672	6.8779	180.26	0.0023	3.7156	3.8055
8	68->72	0.65208	7.0263	176.46	0.0082	-14.3999	-14.0138
9	64->71	0.54871	7.0697	175.37	0.0532	-15.4313	-17.6306
	65->71	0.37541					
10	61->71	0.39248	7.0937	174.78	0.0060	12.1006	12.9672
	62->71	0.29134					
	64->71	0.27446					
11	66->72	0.45204	7.4099	167.32	0.0025	-0.1743	-0.2826
	67->72	-0.27328					
12	61->71	0.37219	7.4380	166.69	0.0058	2.1785	2.4513
	62->71	-0.23067					
	66->72	-0.28386					
	67->72	0.22768					
13	66->72	0.3351	7.5867	163.42	0.0026	-9.7028	-9.4881
	67->72	0.58716					
14	62->71	0.28071	7.6004	163.13	0.0031	1.2949	0.6805
	63->71	0.2748					
	65->71	0.46891					
15	62->71	-0.34356	7.7739	159.49	0.0017	-2.6315	-2.6541

	63->71	0.44368					
16	61->72	0.27395	7.7870	159.22	0.0023	1.7129	1.563
	62->72	0.34816					
	65->72	-0.29708					
17	60->71	0.53932	8.0196	154.60	0.0017	-2.4198	-1.5177
18	61->72	0.41879	8.1060	152.95	0.0100	3.9082	4.5183
	65->72	0.3695					
19	55->71	0.49084	8.1137	152.81	0.0008	-1.004	-1.2057
	56->71	0.28854					
	65->72	-0.24013					
20	64->72	0.59309	8.1626	151.89	0.0117	6.4928	5.6228
	65->72	0.24034					
21	67->73	-0.24343	8.2417	150.44	0.0529	-59.3197	-56.2769
	70->73	-0.22811					
	70->74	0.34933					
22	67->73	0.34591	8.2551	150.19	0.0608	74.8317	77.6804
	67->75	-0.28007					
	70->74	0.30743					
23	59->71	0.24199	8.3059	149.27	0.0388	-0.3746	-1.0344
	69->74	0.44562					
24	62->72	0.40231	8.3367	148.72	0.0222	12.0592	13.9527
	64->72	-0.24585					
	65->72	0.30838					
25	59->71	0.4054	8.5077	145.73	0.0050	-4.9561	-6.0339
	63->72	0.27091					
	70->74	-0.27763					
26	63->72	0.32611	8.5557	144.91	0.0335	-3.6948	-7.8564
	70->73	-0.29217					
27	63->72	0.36503	8.5666	144.73	0.0049	4.466	2.6958
	70->73	0.3251					
28	60->72	0.34602	8.7012	142.49	0.0073	20.3501	19.1933
	68->73	0.2789					
	70->73	0.28478					
29	60->72	0.44742	8.7135	142.29	0.0029	9.4203	10.0493
30	69->73	0.44984	8.7714	141.35	0.0029	-6.1589	-6.5222
	69->74	0.22976					
	69->77	-0.24903					

[a] Number of the excited states; [b] Only transitions with contribution over 10.0% were listed; [c] Configuration-interaction coefficient; [d] Excitation energy; [e] Wavelength; [f] Oscillator strength; [g] Rotatory strength in length form (10^{-40} cgs); [h] Rotatory strength in velocity form (10^{-40} cgs).

Table S18. Key transitions, oscillator strengths, and rotatory strengths in the ECD spectrum of conformer (6*R*, 8*S*)-**3c** at the CAM-B3LYP-SCRF/def2-SVP level of theory in MeOH with SMD solvent model.

Num[a]	Transition[b]	CI-coeff[c]	ΔE (eV)[d]	λ (nm)[e]	f[f]	R_{vel}[g]	R_{len}[h]
1	69->71	0.42815	5.0428	245.86	0.0325	-0.4427	-0.4652
	69->72	0.20567					
	70->71	-0.42557					
	70->72	0.28083					
2	66->71	-0.31584	5.4085	229.24	0.2035	83.7814	91.2555
	69->71	0.3821					
	70->71	0.43256					
3	66->71	0.54428	5.4281	228.41	0.0709	-81.2281	-89.0265
	69->71	0.21218					
	70->71	0.25299					
4	69->71	-0.3342	6.3137	196.37	0.6485	-23.8378	-24.072
	70->72	0.60362					
5	69->72	0.62063	6.4806	191.32	0.4455	-11.5842	-12.3602
	70->71	0.22351					
6	68->71	0.63027	6.5415	189.53	0.0867	18.1484	21.979
7	67->71	0.64502	6.9109	179.40	0.0009	-1.1864	-0.6974
8	62->71	-0.40152	7.0299	176.37	0.0202	-22.9871	-25.8493
	64->71	0.41371					
	65->71	0.24258					
9	62->71	0.23821	7.0785	175.16	0.0364	12.3851	15.7323
	65->71	0.61076					
10	68->72	0.61731	7.1501	173.40	0.0062	10.3936	9.4088
11	61->71	0.27997	7.2835	170.23	0.0045	-6.8935	-7.8544
	62->71	0.26464					
	63->71	0.28515					
	64->71	0.34704					
12	66->72	0.41591	7.3892	167.79	0.0042	-5.2773	-4.2441
	67->72	0.43262					
	68->72	0.286					
13	66->72	-0.39821	7.5656	163.88	0.0045	-6.7171	-7.8926
	67->72	0.48947					
14	61->71	0.39271	7.6529	162.01	0.0047	7.44	7.1019
	63->71	0.22174					
	64->71	-0.36477					
15	61->72	0.2007	7.7474	160.03	0.0041	-4.5856	-4.2964
	62->72	-0.3797					
	64->72	0.42838					

16	61->71	-0.30896	7.7894	159.17	0.0005	-0.9116	-1.2698
	63->71	0.53598					
17	55->71	0.34307	8.0368	154.27	0.0012	-0.0958	-0.1303
	62->72	0.26616					
	63->72	0.23946					
18	67->73	0.37678	8.0494	154.03	0.0010	-3.9039	-2.3557
	68->73	-0.35026					
19	55->71	0.29966	8.0825	153.40	0.0062	-1.6125	-1.4246
	60->71	-0.23656					
	63->72	-0.24716					
20	55->71	0.30905	8.1543	152.05	0.0044	-1.6597	-3.3307
	59->71	0.25947					
	60->71	0.2471					
	65->72	0.37302					
21	55->71	-0.23771	8.1677	151.80	0.0075	2.2573	3.5092
	65->72	0.51578					
22	69->74	-0.20721	8.2227	150.78	0.1112	-9.1812	-9.8108
	70->73	-0.26019					
	70->74	0.48171					
23	62->72	-0.26821	8.3318	148.81	0.0097	24.7542	19.1059
	63->72	0.38155					
	64->72	-0.34043					
24	69->73	-0.22045	8.3569	148.36	0.0358	-8.9503	-8.5993
	69->74	0.46665					
25	59->71	0.40006	8.4355	146.98	0.0091	24.91	19.4661
	60->71	-0.33932					
	61->71	-0.20401					
26	60->71	-0.20414	8.4793	146.22	0.0085	9.9069	5.404
	68->76	0.20869					
27	61->72	0.51211	8.5515	144.98	0.0333	7.8379	8.4721
	63->72	-0.22241					
	64->72	-0.23127					
28	70->73	0.51745	8.6036	144.11	0.0016	-2.2021	-5.4535
	70->74	0.30794					
29	56->71	0.34684	8.6491	143.35	0.0014	-1.3322	-0.6764
	58->71	-0.34067					
	60->71	-0.21067					
30	56->71	-0.26967	8.7467	141.75	0.0020	-5.576	-6.7973
	57->71	0.37533					
	58->71	-0.32832					

[a] Number of the excited states; [b] Only transitions with contribution over 10.0% were listed; [c] Configuration-interaction coefficient; [d] Excitation energy; [e] Wavelength; [f] Oscillator strength; [g] Rotatory strength in length form (10^{-40} cgs); [h] Rotatory strength in velocity form (10^{-40} cgs).

Table S19. Key transitions, oscillator strengths, and rotatory strengths in the ECD spectrum of conformer (6*R*, 8*S*)-**3d** at the CAM-B3LYP-SCRF/def2-SVP level of theory in MeOH with SMD solvent model.

Num[a]	Transition[b]	CI-coeff[c]	ΔE (eV)[d]	λ (nm)[e]	f[f]	R_{vel}[g]	R_{len}[h]
1	69->71	0.472	5.0570	245.17	0.0261	0.5075	0.5862
	70->71	-0.35861					
	70->72	0.30922					
2	69->71	0.37239	5.4100	229.17	0.2738	-41.4755	-45.4942
	70->71	0.54159					
3	66->71	0.63761	5.4342	228.15	0.0084	32.9081	35.7356
4	69->71	-0.34535	6.2940	196.99	0.5942	-11.5093	-11.3683
	70->72	0.595					
5	68->71	0.47359	6.4364	192.63	0.2451	61.715	63.7798
	69->72	-0.4601					
6	68->71	0.47956	6.4810	191.31	0.2977	-14.7023	-14.2133
	69->72	0.46269					
7	67->71	0.66619	6.8417	181.22	0.0105	-2.1477	-2.1827
8	60->71	0.23858	7.0286	176.40	0.0007	1.2556	1.0696
	63->71	0.39237					
	64->71	0.23135					
	65->71	-0.25501					
9	64->71	0.51995	7.0649	175.49	0.0576	-4.6988	-6.7097
	65->71	0.44609					
10	68->72	0.685	7.1412	173.62	0.0053	-1.1645	-1.7993
11	66->72	0.5941	7.3182	169.42	0.0042	3.1668	3.5923
12	61->71	-0.22424	7.3811	167.98	0.0050	9.6959	10.153
	62->71	0.3311					
	63->71	-0.2665					
	64->71	0.24916					
	65->71	-0.29431					
13	67->72	0.66326	7.4170	167.16	0.0218	-36.6543	-38.1341
14	61->71	0.41107	7.6684	161.68	0.0072	-2.2906	-1.8543
	62->71	-0.25484					
	65->71	-0.29022					
15	60->72	0.23017	7.8177	158.59	0.0037	2.854	2.6955
	62->72	-0.24938					
	63->72	0.38795					

	64->72	0.22522					
	66->72	-0.24995					
16	58->71	-0.24014	7.9513	155.93	0.0013	1.9305	2.2085
	59->71	0.2442					
	60->71	0.47071					
17	61->71	0.29848	7.9796	155.38	0.0136	12.2161	11.6904
	62->71	0.39364					
	63->71	0.28185					
18	62->72	-0.24291	8.0216	154.56	0.0083	6.982	8.3893
	65->72	0.49985					
19	56->71	0.52914	8.1173	152.74	0.0030	-2.3438	-1.5855
20	64->72	0.40151	8.1492	152.14	0.0323	13.0949	14.995
	70->73	-0.3579					
21	64->72	0.36104	8.2059	151.09	0.0204	-13.2469	-18.1305
	70->73	0.33937					
22	69->73	0.31155	8.3215	148.99	0.0266	10.6662	10.0859
	69->74	-0.27426					
	70->74	-0.28258					
23	61->72	0.32159	8.3462	148.55	0.0347	-25.1359	-21.0188
	70->74	0.29251					
24	61->72	0.27308	8.4126	147.38	0.0627	-28.9498	-24.1861
	69->74	0.33344					
25	70->73	0.3168	8.5746	144.60	0.0092	2.4853	7.0256
	70->74	0.31698					
26	60->72	0.40447	8.6026	144.12	0.0032	-14.4041	-12.7825
	63->72	-0.36966					
27	57->71	0.34934	8.6420	143.47	0.0030	5.2427	5.6081
	59->71	-0.34894					
	60->71	0.26455					
28	57->71	0.37533	8.6693	143.02	0.0069	7.0897	7.4244
	59->71	0.33536					
	61->71	-0.22746					
29	69->74	0.26964	8.6922	142.64	0.0070	-6.6008	-0.3352
30	61->72	0.32167	8.7145	142.27	0.0064	10.058	10.337
	62->72	0.40866					
	63->72	0.22382					

[a] Number of the excited states; [b] Only transitions with contribution over 10.0% were listed; [c] Configuration-interaction coefficient; [d] Excitation energy; [e] Wavelength; [f] Oscillator strength; [g] Rotatory strength in length form (10^{-40} cgs); [h] Rotatory strength in velocity form (10^{-40} cgs).

Table S20. Crystal data and structure refinement for arthrinin E (**1**).

Identification code	arthrinin E
Empirical formula	$C_{15}H_{26}O_3$
Formula weight	254.36
Temperature/K	100.(2)
Crystal system	trigonal
Space group	$P3_221$
a/Å	8.8861(2)
b/Å	8.8861(2)
c/Å	31.7909(6)
α/°	90
β/°	90
γ/°	120
Volume/Å^3	2173.98(11)
Z	6
ρ_{calc}g/cm^3	1.166
μ/mm^{-1}	0.629
F(000)	840.0
Crystal size/mm^3	0.260 × 0.250 × 0.250
Radiation	Cu Kα (λ = 1.54178)
2Θ range for data collection/°	8.34 to 144.68
Index ranges	$-10 \le h \le 10$, $-10 \le k \le 10$, $-39 \le l \le 39$
Reflections collected	29180
Independent reflections	2838 [R_{int} = 0.0291, R_{sigma} = 0.0139]
Data/restraints/parameters	2838/0/170
Goodness-of-fit on F^2	1.142
Final R indexes [I>=2σ (I)]	R_1 = 0.0351, wR_2 = 0.0969
Final R indexes [all data]	R_1 = 0.0353, wR_2 = 0.0970
Largest diff. peak/hole / e Å^{-3}	0.22/-0.32
Flack parameter	0.07(3)

Table S21. Fractional Atomic Coordinates (×10^4) and Equivalent Isotropic Displacement Parameters ($Å^2$×10^3) for arthrinin E. U_{eq} is defined as 1/3 of of the trace of the orthogonalised U_{IJ} tensor.

**Atom**	***x***	***y***	***z***	**U(eq)**
O1	6545.6(19)	6731(2)	5430.8(4)	23.9(3)
O2	14193.3(19)	6748(2)	5991.6(5)	31.3(4)
O3	7346(2)	1691(2)	6575.7(5)	36.1(4)
C1	10585(3)	9610(3)	5519.6(7)	27.1(4)
C2	10158(2)	8129(2)	5826.4(5)	18.1(4)
C3	8965(2)	6311(2)	5637.5(5)	14.6(4)
C4	7012(2)	5605(2)	5646.8(5)	16.3(4)
C5	6125(3)	3795(3)	5459.1(6)	21.2(4)
C6	6454(2)	2531(2)	5717.5(6)	19.6(4)
C7	6233(3)	1039(3)	5432.5(7)	31.1(5)
C8	11798(2)	8027(3)	5927.3(6)	20.2(4)
C9	11208(2)	6158(2)	6081.2(5)	17.3(4)
C10	11236(3)	6067(3)	6567.3(6)	23.0(4)
C11	12412(2)	5558(3)	5893.6(6)	21.9(4)
C12	9374(2)	5094(2)	5890.5(5)	14.5(4)
C13	8216(2)	3404(2)	5937.8(5)	17.2(4)
C14	8496(3)	2208(3)	6223.9(6)	22.7(4)
C15	9365(3)	8423(3)	6224.8(6)	24.7(4)

Table S22. Anisotropic Displacement Parameters ($Å^2$×10^3) for arthrinin E. The Anisotropic displacement factor exponent takes the form: $-2\pi^2[h^2a^{*2}U_{11}+2hka^*b^*U_{12}+\ldots]$.

**Atom**	**U_{11}**	**U_{22}**	**U_{33}**	**U_{12}**	**U_{13}**	**U_{23}**
O1	28.7(8)	29.3(8)	22.2(7)	20.9(6)	-4.9(6)	2.5(6)
O2	15.1(7)	37.5(9)	43.5(9)	14.7(7)	4.0(6)	4.9(7)
O3	37.6(9)	51.7(11)	27.3(8)	28.5(8)	12.2(7)	20.0(7)
C1	30.9(11)	19.0(9)	30.8(10)	12.1(9)	2.0(8)	7.1(8)
C2	20.2(9)	15.5(9)	18.4(8)	8.7(7)	0.0(7)	0.9(7)
C3	17.6(9)	16.6(8)	11.7(7)	10.2(7)	1.7(6)	1.8(7)
C4	18.4(9)	21.1(9)	13.7(7)	13.1(7)	0.4(6)	3.2(6)
C5	19.2(9)	22.5(10)	19.7(8)	8.8(8)	-3.6(7)	-0.9(7)
C6	18.6(9)	16.9(9)	20.9(8)	7.0(8)	4.0(7)	1.0(7)
C7	32.5(11)	20.2(10)	34.2(11)	8.4(9)	3.1(9)	-5.7(8)

C8	16.7(9)	20.4(9)	20.7(9)	7.2(8)	0.1(7)	1.8(7)
C9	16.1(9)	22.0(9)	15.3(8)	10.7(7)	0.4(6)	2.3(7)
C10	21.7(9)	34.0(11)	16.4(8)	16.2(8)	0.0(7)	2.8(7)
C11	18.8(9)	29.2(10)	21.0(9)	14.5(9)	1.6(7)	2.2(8)
C12	15.6(8)	19.8(9)	12.3(7)	11.9(7)	2.1(6)	1.2(6)
C13	19.7(9)	19.4(9)	16.7(8)	13.0(8)	3.1(7)	1.3(7)
C14	28.0(10)	20.0(9)	25.2(9)	15.7(8)	6.9(8)	6.5(7)
C15	31.3(11)	24.9(10)	22.1(9)	17.1(9)	-0.3(8)	-5.7(8)

Table S23. Bond Lengths for arthrinin E.

Atom	Atom	Length/Å	Atom	Atom	Length/Å
O1	C4	1.434(2)	C5	C6	1.533(3)
O2	C11	1.431(2)	C6	C13	1.526(3)
O3	C14	1.427(2)	C6	C7	1.535(3)
C1	C2	1.526(3)	C8	C9	1.550(3)
C2	C15	1.534(3)	C9	C11	1.535(3)
C2	C8	1.538(3)	C9	C12	1.541(3)
C2	C3	1.544(3)	C9	C10	1.549(2)
C3	C4	1.522(2)	C12	C13	1.338(3)
C3	C12	1.531(2)	C13	C14	1.512(3)
C4	C5	1.516(3)			

Table S24. Bond Angles for arthrinin E.

Atom	Atom	Atom	Angle/˚	Atom	Atom	Atom	Angle/˚
C1	C2	C15	108.55(16)	C2	C8	C9	107.80(15)
C1	C2	C8	110.49(16)	C11	C9	C12	109.92(15)
C15	C2	C8	111.56(15)	C11	C9	C10	109.69(15)
C1	C2	C3	113.48(15)	C12	C9	C10	113.60(15)
C15	C2	C3	111.45(16)	C11	C9	C8	109.18(15)
C8	C2	C3	101.24(14)	C12	C9	C8	102.79(14)
C4	C3	C12	108.90(15)	C10	C9	C8	111.43(16)
C4	C3	C2	117.94(14)	O2	C11	C9	111.38(17)
C12	C3	C2	104.67(14)	C13	C12	C3	122.01(17)
O1	C4	C5	111.95(15)	C13	C12	C9	128.96(16)
O1	C4	C3	111.77(15)	C3	C12	C9	108.97(15)

C5	C4	C3	108.03(15)	C12	C13	C14	123.49(17)
C4	C5	C6	112.25(14)	C12	C13	C6	122.51(16)
C13	C6	C5	113.33(15)	C14	C13	C6	113.97(16)
C13	C6	C7	111.47(16)	O3	C14	C13	110.76(16)
C5	C6	C7	109.13(16)				

Table S25. Hydrogen Bonds for arthrinin E.

D	H	A	d(D-H)/Å	d(H-A)/Å	d(D-A)/Å	D-H-A/°
O1	H24	O1[1]	0.84	1.92	2.754(3)	171.0
O2	H25	O1[2]	0.84	1.92	2.753(2)	170.6
O3	H26	O2[3]	0.84	2.05	2.782(2)	145.0

[1]+Y,+X,1-Z; [2]1+X,+Y,+Z; [3]-Y+X,1-Y,4/3-Z

Table S26. Torsion Angles for arthrinin E.

A	B	C	D	Angle/°	A	B	C	D	Angle/°
C1	C2	C3	C4	-84.4(2)	C8	C9	C11	O2	55.37(19)
C15	C2	C3	C4	38.5(2)	C4	C3	C12	C13	27.0(2)
C8	C2	C3	C4	157.21(15)	C2	C3	C12	C13	153.94(16)
C1	C2	C3	C12	154.39(16)	C4	C3	C12	C9	-150.32(14)
C15	C2	C3	C12	-82.71(18)	C2	C3	C12	C9	-23.33(18)
C8	C2	C3	C12	36.01(17)	C11	C9	C12	C13	67.4(2)
C12	C3	C4	O1	177.34(14)	C10	C9	C12	C13	-56.0(2)
C2	C3	C4	O1	58.3(2)	C8	C9	C12	C13	-176.49(18)
C12	C3	C4	C5	-59.07(17)	C11	C9	C12	C3	-115.60(16)
C2	C3	C4	C5	-178.06(14)	C10	C9	C12	C3	121.07(17)
O1	C4	C5	C6	-173.61(15)	C8	C9	C12	C3	0.54(17)
C3	C4	C5	C6	62.90(19)	C3	C12	C13	C14	-172.95(16)
C4	C5	C6	C13	-31.2(2)	C9	C12	C13	C14	3.7(3)
C4	C5	C6	C7	-156.04(17)	C3	C12	C13	C6	4.7(3)
C1	C2	C8	C9	-157.51(15)	C9	C12	C13	C6	-178.58(16)
C15	C2	C8	C9	81.65(19)	C5	C6	C13	C12	-2.9(2)
C3	C2	C8	C9	-36.99(17)	C7	C6	C13	C12	120.7(2)
C2	C8	C9	C11	139.58(15)	C5	C6	C13	C14	174.97(15)
C2	C8	C9	C12	22.91(17)	C7	C6	C13	C14	-61.5(2)
C2	C8	C9	C10	-99.10(17)	C12	C13	C14	O3	110.9(2)

C12	C9	C11	O2	167.41(15)	C6	C13	C14	O3	-66.9(2)
C10	C9	C11	O2	-67.0(2)					

Table S27. Hydrogen Atom Coordinates (Å×10^4) and Isotropic Displacement Parameters (Å^2×10^3) for arthrinin E.

Atom	*x*	*y*	*z*	U(eq)
H24	6521.69	6557.63	5170.52	36
H25	14835.98	6618.91	5819.02	47
H26	7830.9	2391.22	6774.94	54
H1	11470.88	10709.99	5643.21	41
H8	9532.19	9669.04	5462.2	41
H7	11025.38	9401.18	5256.41	41
H21	9321.85	6315.12	5339	18
H6	6639.8	5514.3	5946.99	20
H5	6556.44	3854.34	5168.94	25
H4	4858.82	3348.31	5443.57	25
H22	5537.65	2019.46	5939.95	24
H3	7195.97	1476.98	5231.12	47
H23	5130.74	563.78	5279.73	47
H2	6231.94	123.91	5604.84	47
H9	12486.91	8879.78	6148.7	24
H17	12531.04	8294.16	5672.89	24
H11	10267.54	6166	6683.47	35
H10	12336.49	7022.47	6673.35	35
H12	11122.17	4955.15	6652.82	35
H13	12085.84	4394.73	6005.96	26
H14	12263.32	5458.32	5584.37	26
H15	8292.73	1164.91	6064.62	27
H16	9713.36	2810.3	6324.48	27
H20	10222.8	9505.78	6360.82	37
H18	9027.5	7449.16	6418.94	37
H19	8337.55	8502.06	6149.04	37
